# Intrinsically disordered regions of Src and Abl kinases contribute to membrane binding

**DOI:** 10.1016/j.jbc.2026.113614

**Published:** 2026-09-30

**Authors:** Svenja de Buhr, Julia P. Steringer, Irem Demir, Saber Boushehri, Ankita Ray, Christina Goss, Andrea M. Adler, Hans-Michael Müller, Rabea Goetz, Camilo Aponte-Santamaria, Britta Brügger, David Alsteens, Walter Nickel, Frauke Gräter

**Affiliations:** 1 Interdisciplinary Center for Scientific Computing, Heidelberg University, Heidelberg, Germany; 2 Heidelberg University Biochemistry Center, Heidelberg, Germany; 3 Louvain Institute of Biomolecular Science and Technology, Université Catholique de Louvain, Louvain-la-Neuve, Belgium; 4 Biomolecular Mechanics, Max Planck Institute for Polymer Research, Mainz, Germany; 5 School of Mathematical and Physical Sciences, University of Sheffield, Sheffield, United Kingdom

**Keywords:** Abl kinase, myristoylation, membrane recruitment, mechanokinases, intrinsically disordered proteins, phosphatidylinositol-4,5-bisphosphate, plasma membrane, Src kinase

## Abstract

Membrane targeting regulates protein localization and function. Abl and Src kinase feature an N-terminal myristoylation site followed by an intrinsically disordered region (IDR) of positive net charge, suggesting these regions contribute to membrane targeting. We investigated the interplay of myristoylation and IDR binding to phosphatidylinositol-4,5-bisphosphate (PIP_2_)-containing membranes using atomistic equilibrium and non-equilibrium molecular dynamics (MD) simulations. After having established Charmm36m-TIP4P-fb as a force field combination of choice for the challenging IDR-membrane systems of interest, we assessed the binding propensities of Abl and Src’s IDRs to lipid bilayers, and observed strong binding only when PIP_2_ was present, as also quantified by significant increases in detachment forces and unbinding free energies when including 10 mol% PIP_2_. Interactions were mediated by saltbridges between the IDR’s basic residues and PIP_2_ headgroups, and largely unaffected by cholesterol. On this basis, we designed Src and Abl mutants with deficient PIP_2_ binding, and confirmed impaired membrane binding using liposomal-binding assays and atomic force microscopy experiments. Simulations and experiments of further variants corroborated that both a myristoylation site and the charged IDR are required for firm PIP_2_-membrane interactions. Our results establish myristoylated IDRs in kinases - and potentially beyond - as important regulators of PIP_2_-specific membrane targeting.

Membrane binding plays a critical role in regulating protein function. Many proteins, notably kinases pivotal in signal transduction, are regulated not only by molecular mechanisms such as conformational control or post-translational modification, but also by their sub-cellular localization. Membrane association enables these proteins to access membrane-bound signaling complexes or substrates (1, 2). Importantly, the process of membrane targeting can extend beyond mere localization — affecting protein conformation and function (3). For example, focal adhesion kinase (FAK) demonstrates that membrane interactions can induce conformational shifts that modulate activity (4, 5).

Two other prominent examples are the tyrosine kinases Abl and Src. Both kinases are myristoylated at their disordered N-terminal regions (Fig. 1*A*), a modification that facilitates membrane targeting and underscores the regulatory potential of intrinsically disordered regions (IDRs) as flexible tethers. In the case of Src, electrostatic interactions between positively charged residues and acidic membrane lipids are known to contribute significantly to its membrane binding (6, 7, 8). Recent findings reveal that Abl similarly engages in membrane binding, possibly through mechanisms analogous to those of Src (9). Similarly, HIV-1 Nef has been reported to bind to membranes *via* a positively charged myristoylated N-terminal IDR (10, 11). Amino acid substitutions within an arginine cluster (R17-R22) of its IDR are known to reduce membrane association dramatically and are completely abrogated when lysine 4 and 7 are substituted in addition (12). HIV-1 Nef membrane binding has been proposed to represent a biphasic process that requires negatively charged lipids for the initial contact mediated by the arginine-rich region. N-myristoylation and hydrophobic residues then insert into the lipid bilayer, stabilizing membrane binding through the formation of an amphipathic helix (11).

**Figure 1:**
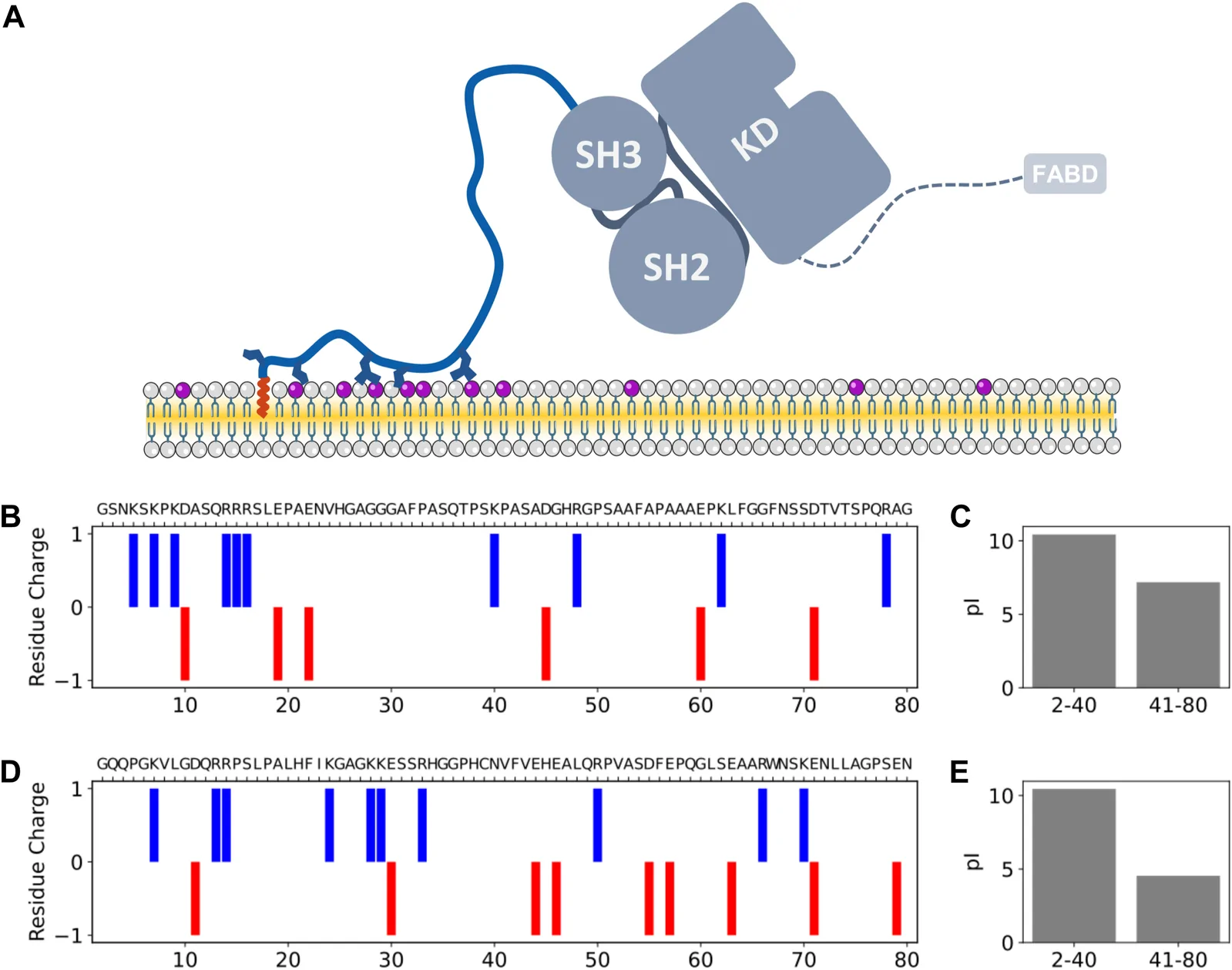
**Abl and Src both feature basic, N-myristoylated and disordered N-terminal linkers.***A*, schematic showing the arrangement of catalytic kinase domain and regulatory SH2 and SH3 domains as well as the myristoylated (*red*), disordered N-terminal sequence (*blue*) of Abl and Src. Abl additionally possesses a C-terminal disordered region and F-actin binding domain. Sequence comparison of Src’s (*B and C*) and Abl’s (*D and E*) N-terminal disordered regions showing charges per residue (*B and D*) and the isoelectric point of the first (residues 2-40) and second half (41–80) of their disordered region. See Figs. S5 and S6 for the full sequence alignments.

Phosphatidylinositol-4,5-bisphosphate (PIP_2_) is an acidic, *i.e.* negatively charged, lipid and a common mediator of membrane interactions with clusters of basic residues in proteins. As a key signalling lipid found in the inner leaflet of the plasma membrane, it is enriched at focal adhesion sites and specifically anchors proteins with lysine or arginine clusters, such as FAK, to the membrane (4, 13, 14, 15). Both experimental and computational studies suggest that local concentrations in the range of ∼5 to 10 mol% are plausible within such confined regions (16, 17, 18). These novel insights prompt the hypothesis that myristoylated IDRs may represent a template common among signaling proteins, with both the myristoylation as well as IDR contributing to targeting these proteins to membranes enriched in acidic lipids. Interestingly, Src kinase and Abl kinase are both known to localize to focal adhesions and other actin-rich membrane regions, where they interact with adhesion-associated proteins and participate in signal transduction (19, 20). In these environments, membrane composition is actively regulated and PIP_2_-enriched (15), which might aid the anchoring of the N-terminal positively charged IDR of the two kinases. We here ask how the myristoylated IDRs of Src and Abl kinase stably bind to membranes. Key questions we address are: How do myristoylated IDRs behave at the membrane interface? How is this interaction influenced by specific lipids, such as PIP_2_ and cholesterol? And what governs their binding strength?

In this work, we employ a combination of molecular dynamics (MD) simulations, biochemical analysis of Src and Abl kinase recruitment to membranes, and atomic force microscopy (AFM) to systematically explore the nature of IDR-membrane interactions. As a starting point for the MD simulations, we assessed the feasibility of currently available force fields to faithfully study IDR-membrane interactions. This is challenged by the fact that canonical protein force fields that have been previously validated for their compatibility with force fields for lipid bilayers fail to reproduce the collapse propensity of IDRs, while IDR-adopted force fields have not yet been systematically tested for systems that also include membranes. We then characterized the membrane binding properties of Src and Abl as model systems, focusing on the interplay of myristoylation, electrostatic and hydrophobic interactions in simulations, to then test our predictions for WT and a computationally designed mutant in biochemical analyses of purified Src and Abl kinase variant proteins using sedimentation of large unilamellar vesicles (LUVs) and AFM on supported lipid bilayers (SLB). Our results indicate that membrane anchorage is stabilized by both the myristoyl modification and the cluster of positively charged residues. PIP_2_ lipids are essential for supporting the electrostatic interactions between the IDRs and the membrane, whereas the inclusion of cholesterol has minimal additional impact.

This study not only elucidates the underlying mechanisms of membrane tethering mediated by IDRs but also paves the way for a broader understanding of how membrane anchoring impacts kinase signaling and function.

## Results

Previous studies have shown that positively charged amino acids close to the myristoyl (Myr) modification site on Src's N-terminal flexible region contribute to stable membrane binding (6, 7, 8). Wondering whether the same applies to Abl, we compared their sequences ((Fig. 1, *B*–*E*). In contrast to the cluster of lysines and arginines from residue 5 to 16 for Src, the positive charges at Abl's N-terminus are more dispersed. Yet, the first half of the Myr-linker displays a pronounced positive net charge, leading to an isoelectric point (pI) very similar to that of Src (pI = 10.43 and pI = 10.45 for Src and Abl, respectively, (Fig. 1, *C* and *E*). This raises the question if charges have to be spatially clustered as in the case of Src or if more dispersed charges are sufficient to support membrane binding of Abl.

For more insight into the interactions of the two kinases with membranes, we performed MD simulations, for which we first had to identify a suitable force-field. The Charmm36m force-field in combination with TIP3P water is popular for the simulations of membranes. It however overestimates protein-protein interactions and thus leads to strongly collapsed ensembles of disordered peptides (21). A widely used force-field for IDPs, Amber99sb∗-ILDNP in combination with TIP4P-D water, on the other hand, is not appropriate for membrane simulations. Since IDP properties are largely influenced by the water model, we decided to test the Charmm36m force-field with various water models. The force-balance method gave rise to two water models, TIP3P-fb and TIP4P-fb, with reparameterized Lennard-Jones terms causing an improved viscosity and dielectric constant compared to TIP3P water (22). This might lead to a better balance of electrostatic and dispersion interactions and thus to enhanced solvation of IDPs. Additionally, they have been validated for the use in membrane MD simulations (23). We tested the two force-balance water models by simulating the first half (MyrGly2-Gly34) or the whole (MyrGly2-Asn79) Abl Myr-linker region. In our tests with the Charmm36m force field, we additionally included TIP3P as a reference for collapsed ensembles, the 'IDP water model' TIP4P-D, and, as a reference for a well represented IDP, the Amber99sb∗-ILDNP force-field with TIP4P-D water. The force-balance water models give rise to improved radius of gyration (R_G_) values compared to TIP3P water ((Fig. 2) and the results of TIP4P-fb in combination with Charmm36m are very similar to Amber99sb∗-ILDNP with TIP4P-D water (mean R_G_ = 2.55 nm and R_G_ = 2.54 nm, respectively). For comparison with experimental results, we simulated the IDR of the *S. cerevisiae* transcription factor Ash1 (residues 420-500), and obtained R_G_ values in agreement with previously published results and with experiment (24) ((Fig. S1). Taken together, Charmm36m together with TIP4P-fb water is a good choice for simulations of combined IDP and membrane systems and was chosen for all subsequent simulations.

**Figure 2:**
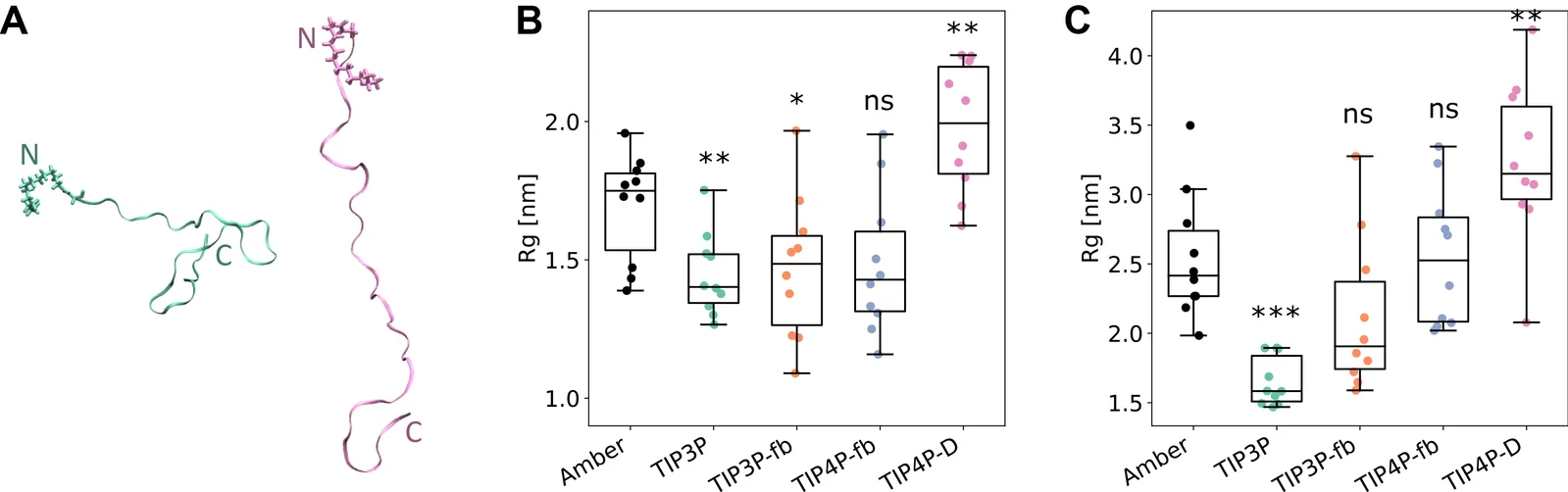
**Validating force field and water combinations for IDP-membrane systems.***A*, exemplary snapshots for Abl MyrGly2-Gly34 simulated with TIP3P (*green*) or TIP4P-D (*pink*) water. Myr is shown in stick representation. Radius of gyration values for (*B*) MyrGly2-Gly34 and (*C*) MyrGly2-Asn79. The peptides were simulated with the Charmm36m force field combined with the indicated water model or with the Amber99SB∗-ILDNP in combination with TIP4P-D water (labeled as Amber). Medians are shown as *central lines*, *boxes* indicate the 25 and 75 percentile, and whiskers represent 1.5 × inter-quartile range. *p*-values of the different water models, obtained using a two-sided independent *t* test, are indicated compared to the Amber force field. ∗∗∗: *p* < 0.001, ∗∗: *p* < 0.01, ∗: *p* < 0.05, ns: not significant.

For exploring peptide membrane interactions and to assess the influence of different charge patterns at the N-termini of Src and Abl (refer to (Fig. 1, *B* and *D*), we simulated the first 34 residues of each kinase, which harbor the myristoylation site and the majority of positively charged residues. We used a pure POPC bilayer to approximate the membrane. For assessing electrostatic interactions, we added 10% PIP_2_ into the membrane leaflet facing the peptides, with or without the addition of 30% cholesterol. The chosen PIP_2_ concentration mimics a strong enrichment of PIP_2_ as for example found at focal adhesion sites (14, 25, 26). We inserted the Myr moiety of pre-equilibrated peptides into the lipid bilayers and let the remaining peptide freely associate to the membrane. The peptides of both kinases interact more closely with the membrane when PIP_2_ is present ((Fig. 3). In contrast to previous findings showing that cholesterol can increase binding by improving head group exposure (27), the addition of cholesterol does not further enhance the interaction, indicating that peptide-PIP_2_ interactions are saturated at 10% PIP_2_. In line with the peptide’s sequence comparison, the most N-terminal residues of Src interact more closely with the membrane upon PIP_2_ addition while residues farther away from the Myr-site remain solvent exposed. In the case of Abl, the whole length of the simulated Myr-linker associates towards the membrane leading to an overall smaller peptide-membrane distance compared to Src. We find Abl’s and Src’s IDR to remain largely disordered, with only very marginal transitions to helices ((Fig. S11), in line with the low very propensity to form (amphipathic) helices predicted based on sequence ((Fig. S12).

**Figure 3:**
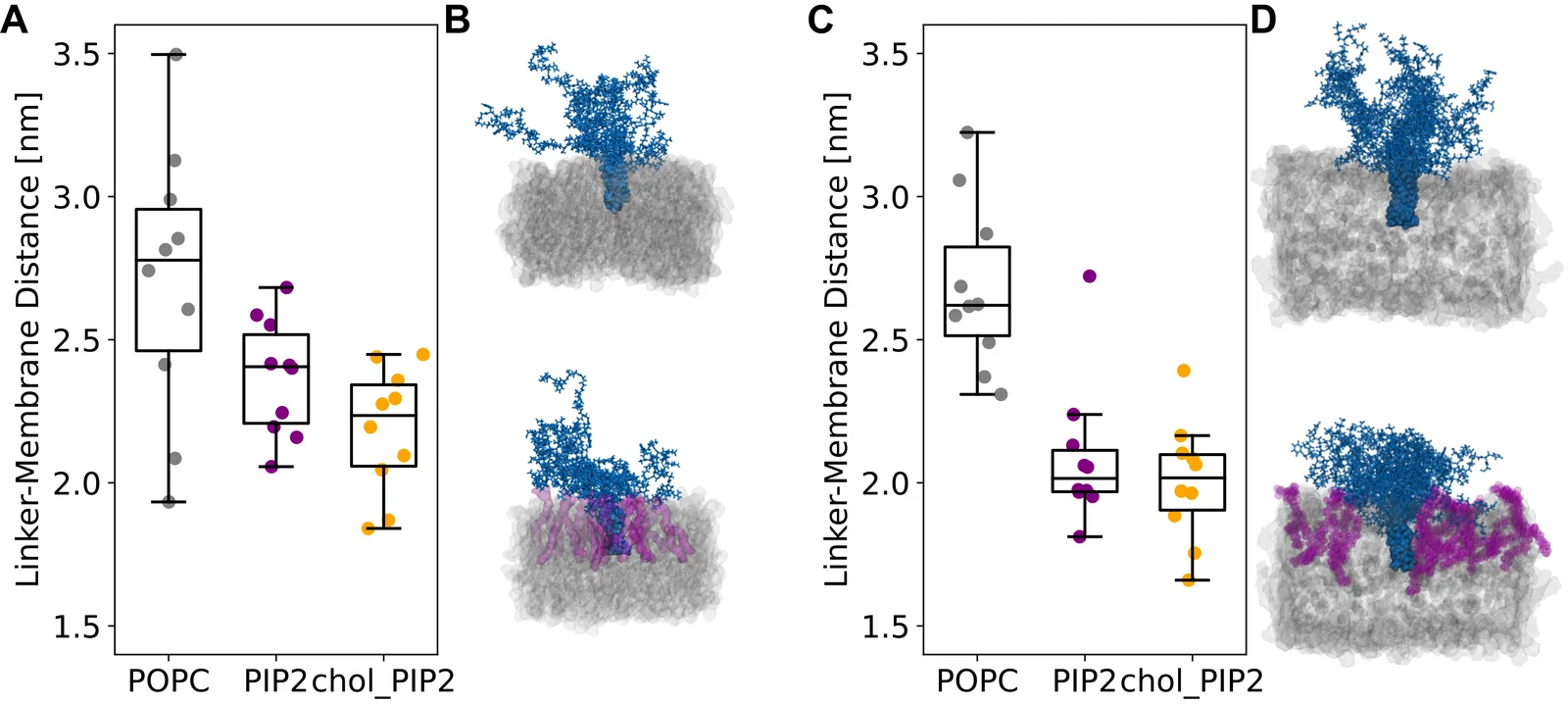
**Distance between membranes and Src (*A and B*) or Abl (*C and D*) N-terminal peptide's center of mass.** Membrane compositions were 100% POPC (*gray*; *B and D upper*), POPC:PIP_2_ 90:10 (*purple*; *B and D lower*), or POPC:PIP_2_:chol 60:10:30. Data is presented as in Figure 2. PIP_2_, phosphatidylinositol-4,5-bisphosphate.

To explore the functional implications of closer membrane interaction of the kinase's N-terminal regions in the presence of PIP_2_, we moved on to determine the detachment force of the IDR from the membrane as well as the free energy of binding using additional umbrella sampling simulations. The IDRs of both Src and Abl kinase require significantly higher forces for unbinding from a PIP_2_ membrane compared to a pure POPC membrane ((Fig. 4*A*) and also show higher binding free energies in the presence of PIP_2_ ((Figs. 4*B* and (S2 for free energy profiles of binding). Again, the presence of cholesterol did not impact the binding strength. Interestingly, the maximal force shown in (Figure 4*A* was reached, when the positively charged regions closest to the myristoylation site of the two peptides finally detached from the negatively charged PIP_2_ head groups. Our data thus suggest that the positively charged residues strongly contribute to PIP_2_-membrane binding. We also observed both proteins to show very similar unbinding forces and free energies, suggesting that the extent of charge concentration along the IDR, when comparing the two cases of Abl with more wide-spread charges and Src with more concentrated charges, does not matter when it comes to PIP_2_-mediated membrane binding. Interestingly, in both equilibrium and pulling MD simulations, the membrane exhibits local curvature changes centered on the myristoylation site ((Fig. S13). However, larger simulation systems would be needed to attenuate periodic boundary effects and to investigate the effect of IDR-membrane interactions on curvature further.

**Figure 4:**
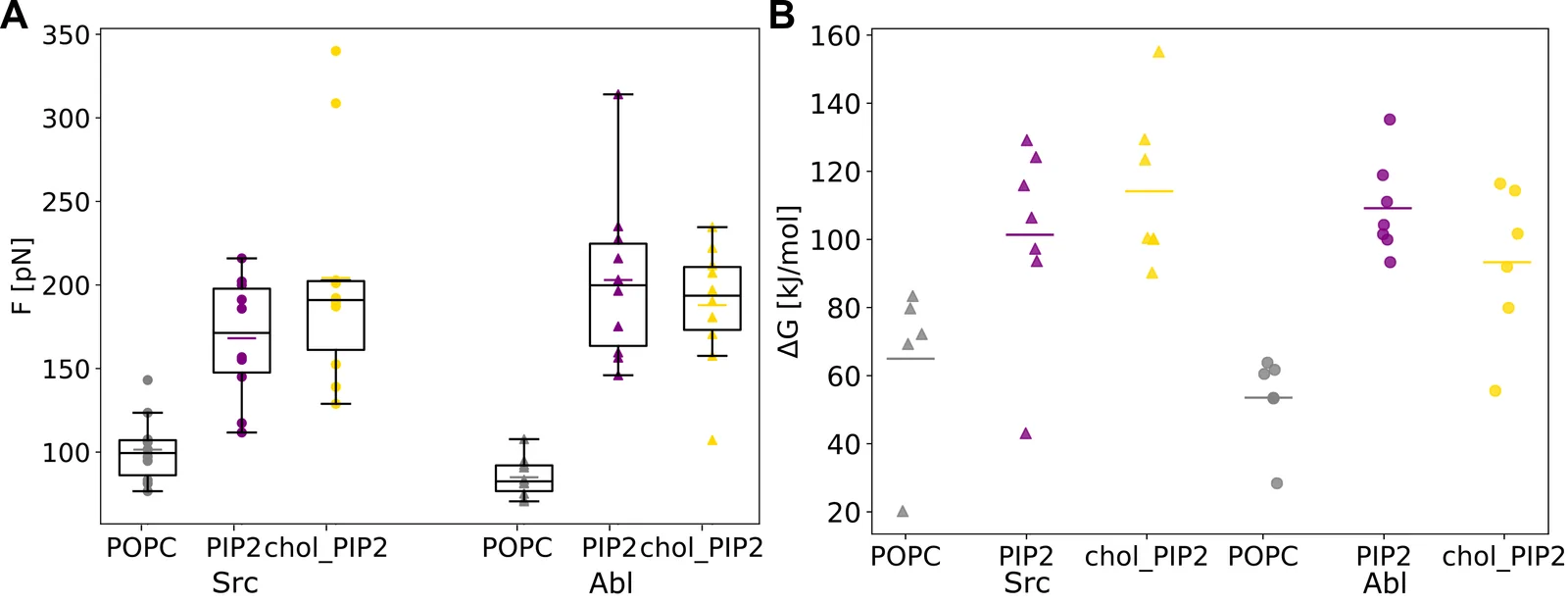
**N-terminal linkers bind in both mechanical and thermodynamic terms more strongly to membranes containing PIP_2_.** Forces (*A*) needed to remove and free energy of unbinding (*B*) Abl and Src N-term peptides from membranes with or without PIP_2_ and cholesterol. The corresponding PMF profiles are shown in Fig. S2. Values from individual replicates are shown as scatters, while the *line* represents the mean. *Box plots* are presented as in Figure 2. PIP_2_, phosphatidylinositol-4,5-bisphosphate.

We next tested these predictions from MD simulations by liposome binding assays of the two kinases. N-terminal myristoylation of Abl and Src WT proteins has been confirmed by MS, (Figs. S7 and (S8. Whereas the N-terminal peptide of Abl G2A (AQQPGK) did not show myristoylation, as expected (MS, (Fig. S9), the N-terminal peptide of Src G2A (ASNK) could not be detected as absence of myristoylation results in a hydrophilic tetrapeptide with insufficient column retention. Following incubation of large unilamellar vesicles with Src or Abl protein variants, respectively, membrane bound material was separated from unbound fraction by centrifugation and analyzed by SDS-PAGE under reducing conditions ((Fig. S10). We find Src binding to membranes, with stronger binding to PIP_2_-containing liposomes, and reduced binding when Myr was absent (G2A mutation, (Figure 5). In contrast to Abl’s well-studied myristoyl binding pocket at the kinase C-terminal lobe (28, 29), a well-defined pocket has not yet been resolved in X-ray structures of Src, but has been previously predicted on the kinase domain based on structural data (30). A mutation designed to block the proposed binding pocket (T459F) indeed leads to a slight increase in PIP_2_-dependent binding, while binding to POPC-liposomes remains as low as for the WT. Overall, this confirms Src to bind to membranes in a PIP_2_- and myristoyl-dependent manner.

**Figure 5:**
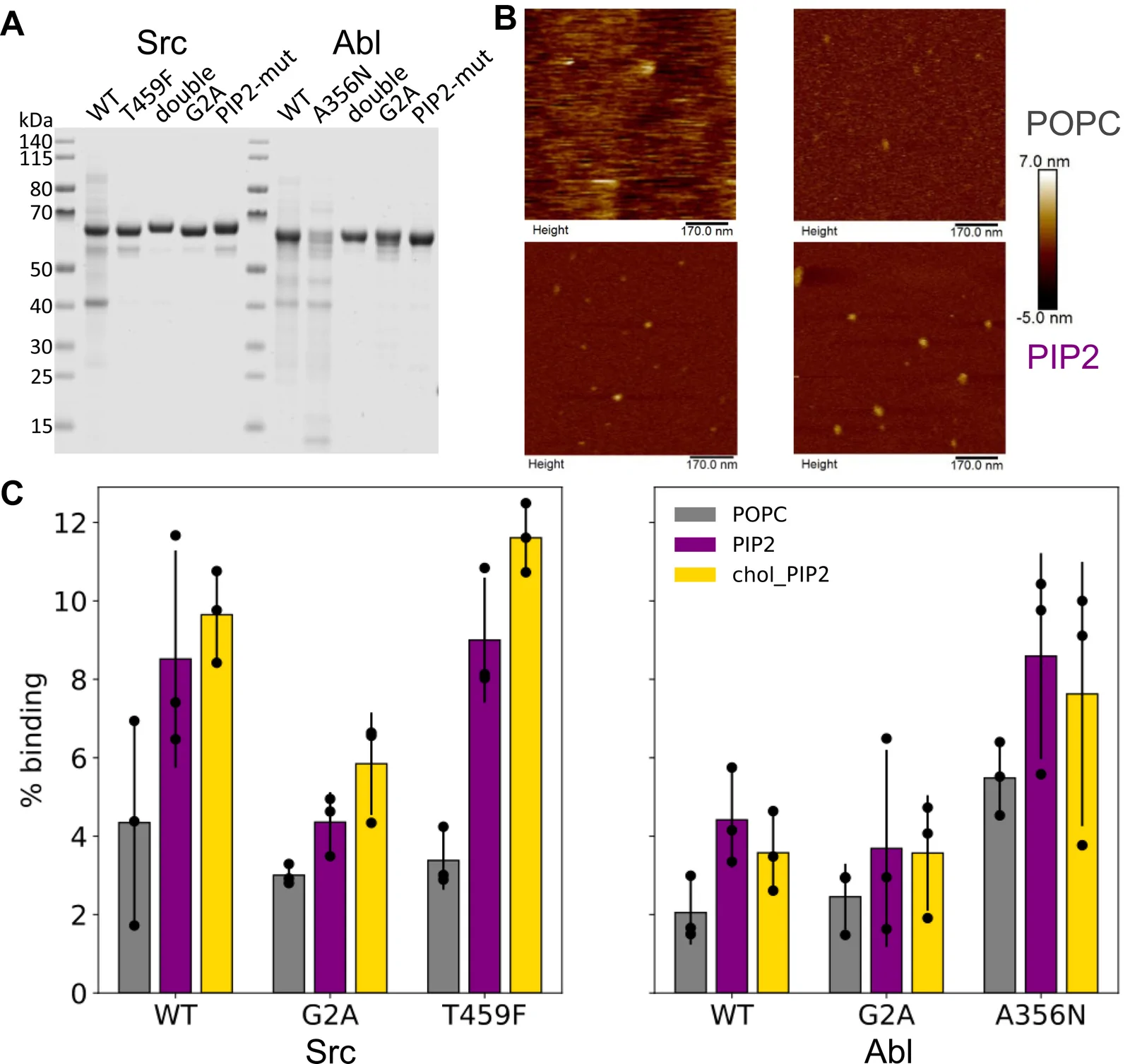
**Binding of Src and Abl to liposomes.***A*, SDS-PAGE analysis of Src and Abl (M1-K531) variant forms indicated. PIP2 refers to mutants in which the positively charged residues on the IDRs were replaced by glutamine. The ‘double’ mutation combines the PIP2 and G2A variants. Purified proteins were analyzed for homogeneity using Coomassie staining. *B*, atomic force microscopy of Src-WT (*left*) or Abl-WT (*right*) on pure POPC (*top*) or POPC:PIP_2_ 90:10 supported lipid bilayers. *C*, biochemical analysis employing large unilamellar vesicles with different lipid compositions: a. 99 mol% POPC + 1 mol% Rhodamine-PE (POPC, *grey bars*), b. 10 mol% PIP_2_ + 89 mol% POPC+ 1 mol% Rhodamine-PE (PIP2, *violet bars*), or c. 10 mol% PIP_2_ + 30 mol% Cholesterol + 59 mol% POPC + 1 mol% Rhodamine-PE (chol_PIP2, *yellow bars*). Mean values with SD of three independent experiments are shown. PIP_2_, phosphatidylinositol-4,5-bisphosphate.

In corresponding experiments for Abl kinase, we find Abl to bind to membranes with overall lower binding propensity compared to Src. While differences between myristoylated and non-myristoylated Abl are minor, a constitutively active mutation that blocks Myr binding to its kinase domain (A356N) (29) leads to stronger liposome binding in a PIP_2_-dependent fashion. We conclude that in the case of Abl kinase, exposure of the myristoylated N-terminus as well as PIP_2_ are required for association of the protein to the membrane. We additionally asked if PIP_2_-mediated membrane binding could be altered by cholesterol (27). We find very minor changes in membrane binding when including 30 mol% cholesterol in the PIP_2_-containing membranes, with minor but systematic increases in case of Src and largely insignificant changes for Abl.

We observed qualitatively similar trends for Src and Abl binding to membranes when analyzing the binding of the proteins to supported bilayers using atomic force microscopy (Fig. 5*B*). WT kinases showed no binding in the absence of PIP_2_, and binding to PIP_2_-membranes, as evident from bright protrusions with several nanometers (ranging from 3 nm to 5 nm) on the lipid bilayers, in line with the protein size expected from their structure (∼3 nm for the folded domains of both Abl and Src kinase) (31, 32).

Having established the important role of PIP_2_ in mediating kinase-membrane interactions, we next analyzed in further detail the nature of the protein-PIP_2_ interaction and the role of the IDR’s positive charges. (Figure 6, *A* and *C* show the contacts along the peptide sequence with membrane lipids. We observe defined peaks in the number of contacts per residue, which correspond to Arg and Lys residues of the IDRs of Src and Abl, respectively. As expected, these contacts are primarily established with the negatively charged PIP_2_ headgroups of the lipid bilayer (Fig. 6, *B* and *D*). We mutated these residues (7 and 6 residues in Abl and Src, respectively) to glutamine, resulting in Abl-K7/R13/R14/K24/K28/K29/R33Q and Src-K5/K7/K9/R14/R15/R16Q (see Figs. S5 and S6 for the full mutant sequences), and asked if protein-PIP_2_ interactions were lost as a consequence. Indeed, compared to the WT counterparts, the strong increase in contacts and binding free energy when moving from POPC-only to PIP_2_-containing membranes is largely lost for the Src and Abl PIP_2_-binding mutants (Fig. 7, *A* and *B*, compare to Figures. 4*A* and 6, *B* and *D*). We could confirm these computational predictions experimentally by liposome binding of the same mutants (Figs. 7*C*, S3 and S4 for respective AFM images). The PIP_2_ dependency of binding is lost in the case of Abl when positively charged residues are mutated. Src PIP_2_ binding only decreased when both positively charged residues and the myristoylation site were mutated. Apparently, other residues of Src, within its IDR or folded domains that are not included in the simulations can rescue the depletion of the basic residues but not of both.

**Figure 6:**
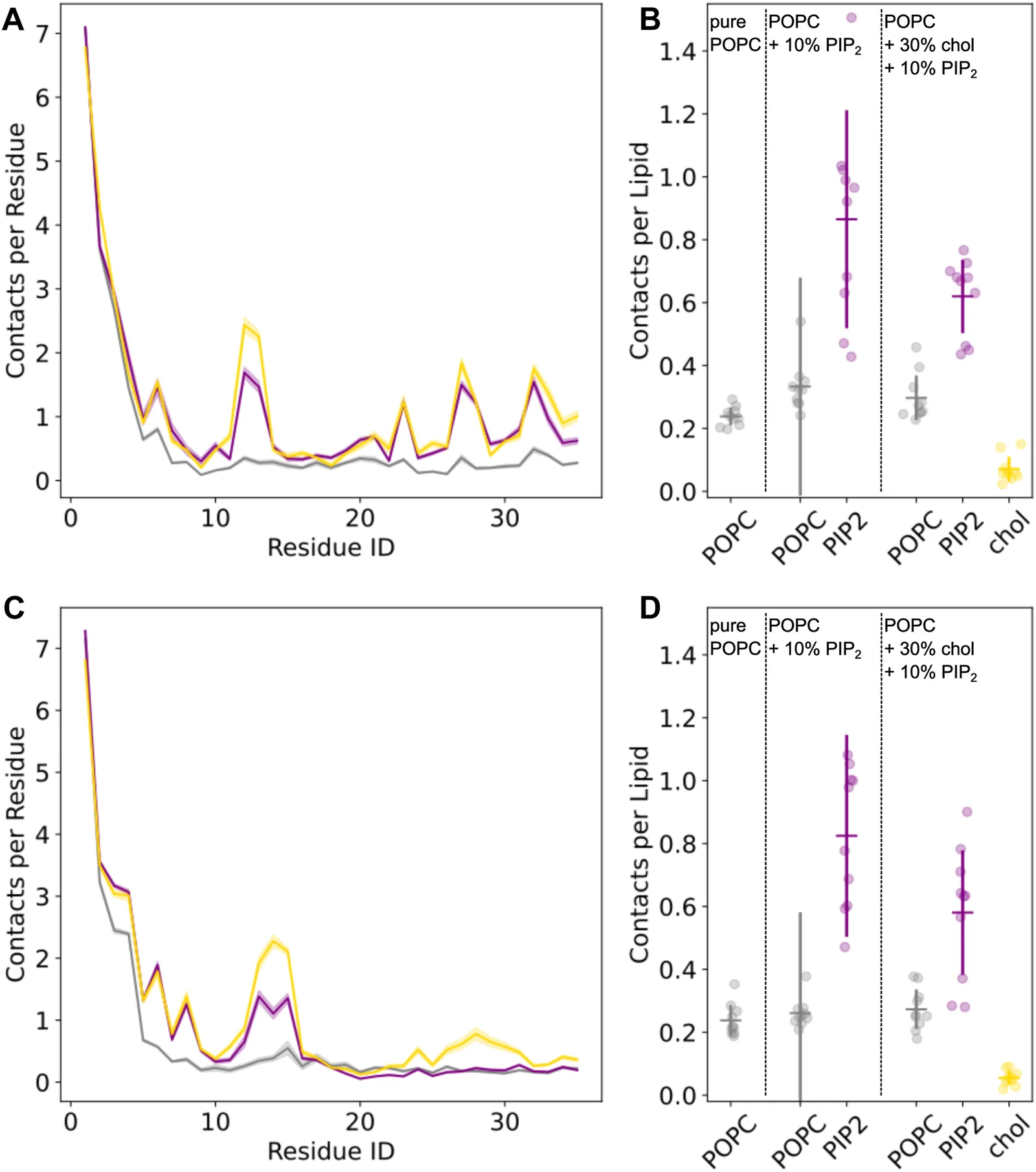
**Basic residues mediate lipid contacts.** Contacts per residue or per lipid for (*A and B*) Src and (*C and D*) Abl. Solid lines in panels *A and C* as well as scatter points in *B and D* represent mean values. SEM is depicted as shades and *error bars* in A/C and B/D, respectively. n = 10.

**Figure 7:**
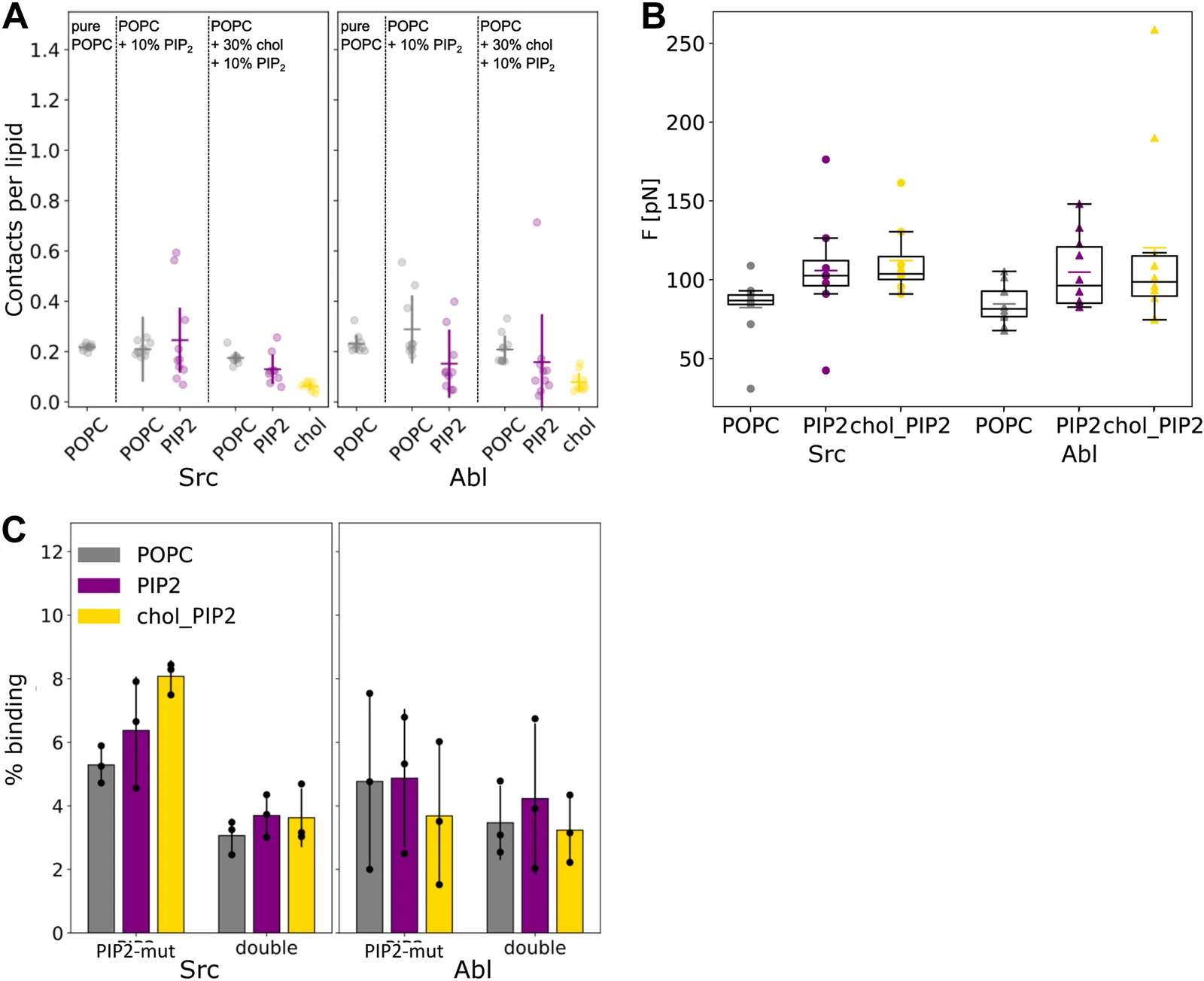
**Mutations of basic residues and removal of myristoylation abolish strong membrane anchoring on PIP2 membranes in simulations and experiment.***A*, contacts per lipid with mutated Src and Abl N-terminal peptides in MD Simulations represented as mean ± SEM and (*B*) forces required to detach these peptides from POPC membranes with and without PIP_2_ or cholesterol. The data is represented as described in Figure 2. *C*, Percent of protein bound to liposomes when N-terminal positively charged residues are changed to glutamine (termed PIP_2_ mutant). The double mutant combines the G2A and PIP_2_ mutant, based on biochemical analysis employing large unilamellar vesicles with different lipid compositions (see Fig. 5 for details). PIP_2_, phosphatidylinositol-4,5-bisphosphate.

## Discussion

Our study combines molecular simulations, binding assays and mutagenesis to elucidate the role of the IDRs of Src and Abl kinases for their interaction with lipid membranes.

One important outcome of our work is the assessment of atomistic protein, membrane and water force fields to study IDR-membrane interactions, which will be of use in future studies. Canonical protein force fields often fail to accurately reproduce the collapse propensity of IDRs, while force fields adapted for IDRs have not been systematically tested with membranes. This study found that the Charmm36m force field paired with TIP4P-fb water is effective. The TIP4P-fb model, derived using the force-balance method, exhibits an improved viscosity and dielectric constant compared to TIP3P water (22, 23), which leads to enhanced solvation of IDPs. The results obtained with Charmm36m/TIP4P-fb were found to be very similar to the established IDP force field combination (Amber99sb∗-ILDNP/TIP4P-D water) in reproducing the R_G_ of the Abl Myr-linker region. This methodological advancement ensures reliable simulation results for similar complex biomolecular systems including membranes and IDRs.

The MD simulations provided substantial insight into the nature of IDR-membrane interactions, revealing a strong dependence on PIP_2_. When the IDR linkers were simulated in isolation (meaning the N-terminal Myr moiety was exposed and not hidden by the kinase domain), the peptides showed significantly closer membrane interaction, higher detachment forces, and increased binding free energies in the presence of PIP_2_ compared to pure POPC membranes. The introduction of 30% cholesterol, however, did not further enhance the interaction, suggesting that the peptide-PIP_2_ interactions were already saturated at the 10% PIP_2_ concentration used.

This strong binding affinity to PIP_2_ is mediated primarily by electrostatic interactions between the basic residues (Arg and Lys) within the IDR and the negatively charged PIP_2_ headgroups. Interestingly, both Src (with concentrated positive charges) and Abl (with more dispersed positive charges) showed very similar unbinding forces and free energies in MD, suggesting that the extent of charge concentration along the IDR may not significantly impact the total PIP_2_-mediated binding strength. In agreement, we recently found PIP_2_ to be crucial for the mechanical resistance of Src membrane binding when using a shorter fragment of the linker (residues 2-19) (6). Src (6, 33) and putatively Abl (34, 35) are involved in the mechanoresponse of cells and our data suggests that PIP_2_-mediated membrane binding is required to attach the kinases firmly enough to allow kinase activation without compromising the membrane anchoring. A sole myristoylation site would not suffice for this purpose (6, 36, 37). Our experiments and simulations are based on 10% PIP_2_ (see Experimental Procedures), and the effect of variations of this concentration on the observed effects remains to be investigated.

Our combined data suggest that both Src and Abl may display a myristoyl-switch mechanism, involving the conditional exposure of the Myr group. Hantschel, *et al.* (2003) (29) have reported Abl to be regulated by a myristoyl/phosphotyrosine switch that involves Myr being buried in a hydrophobic cleft within Abl's kinase domain, thereby stabilizing an autoinhibited state. Thus, Myr has to detach from the kinase for membrane binding. This is consistent with our data and explains the overall low binding propensity in experiments with Abl’s WT. A constitutively active mutation designed to block the internal myristoyl-binding pocket (29) led to strong, PIP_2_-dependent liposome binding. This strongly suggests that for Abl kinase, the Myr moiety must be exposed, along with PIP_2_ availability, for association with the membrane to occur. We have previously shown that Abl and the membrane can compete thermodynamically for Myr binding, highlighting the interplay between kinase regulation and localization (9). This current study adds lipid composition as an extra regulatory layer and underscores the crucial link between membrane binding and kinase activity. It supports a model in which the N-terminal IDRs of Src and Abl act as context-dependent regulatory elements that coordinate conditional Myr exposure through electrostatic interactions between IDR residues and PIP_2_. Furthermore, membrane association enables these pivotal kinases to access membrane-bound signaling complexes or substrates, and membrane interactions can modulate protein conformation and function.

Both Src and Abl are implicated in the mechanoresponse of cells, including roles in cytoskeleton reorganization and focal adhesion maturation (6, 33, 34, 35, 38, 39). The strong dependence on PIP_2_ observed in the MD simulations of isolated linkers confirms that PIP_2_ is crucial for the mechanical resistance of membrane binding. The observation that Abl generally showed lower membrane affinity compared to Src suggests that other contextual factors must regulate membrane interaction, particularly for Abl. In a cellular context, factors such as phosphorylation or the binding of other regulatory proteins might be required to properly expose Myr and bring the positive residues of the IDR into sufficient proximity to the membrane surface, thus regulating the protein's sub-cellular localization and activity. Lipids beyond PIP_2_ and cholesterol, such as negatively charged but monovalent DOPS or sphingomyelin might further modulate the interactions. The IDR’s strong dependence on PIP_2_ highlights a possible coupling mechanism between lipid composition sensing, kinase localization and kinase activation.

We propose that the dual anchoring of IDRs through both myristoylation and clusters of acidic residues is likely a common theme of signalling proteins with both disordered and folded domains. HIV Nef-1 closely follows the same theme (10, 11). A proteome-wide analysis could identify the most common sequence motifs of acidic clusters and the sequence composition typical to such IDRs adjacent to myristoylation sites, to shed further light on the anchoring function of membrane-interacting IDRs and their regulation.

## Experimental procedures

### Molecular dynamics simulations

We used Chimera (40) to build linear structures of the peptides and the Charmm-GUI (41, 42) input generator to add the N-terminal myristoylation and C-terminal NME-caps. Isoelectric points were calculated using the Compute pI tool on the Expasy Web Server (43, 44, 45), while amphipathy was predicted using the AmphipaSeeK method (46). We placed the peptides in triclinic simulation boxes with at least 2 nm distances to all box edges and performed short 20 ns simulations to relax the linear structures. We picked frames from these relaxation simulations as start structures for subsequent production simulations. For membrane generation, we used the membrane builder entity of Charmm-Gui. The pure POPC bilayer consisted of 99 lipid molecules per leaflet, the bilayer with 10% PIP_2_ contained 100 lipids per layer, with 10 molecules of PIP_2_ in the upper leaflet and 90 and 100 molecules of POPC in the upper and lower leaflet, respectively. The membrane with 10% PIP_2_ and 30% cholesterol consisted of 112 POPC and 48 cholesterol molecules in the lower leaflet and 96 POPC, 48 cholesterol and 16 PIP_2_ lipids in the upper leaflet. This composition leads to bilayer sizes with approximately 8 nm edge length. We chose a 10% PIP_2_ concentration, as previous studies have suggested a strong local enrichment of PIP_2_ beyond the 1 to 2% average concentration in the inner leaflet of the plasma membrane (47). This concentration has also been used previously to study FAK-membrane interactions (48, 49).

All simulations were done using Gromacs version 2020.3 and 2022.5 (50, 51) and the Charmm36m force field (March 2019 version) (52, 53, 54) along with TIP4P-fb water (22, 23) if not stated otherwise. After placing peptides and/or lipid bilayer in simulation boxes and solvation, we added 150 mM NaCl for ionic strength and to neutralize charges. Simulation boxes for peptide only simulations were dodecahedral with a size that left at least 2 nm between box edges and the nearest peptide atom. Simulation boxes involving bilayers had a triclinic shape and box widths in xy-direction corresponded to the size of the membrane and box heights were approximately 12 nm for equilibrium simulations and 20 nm during force-probe and umbrella sampling simulations. The steepest descent algorithm with a step size of 0.01 nm was applied for energy minimization until the force on all atoms was lower than the threshold of 1000 kJ/mol/nm. During a 100 ps long simulation in the NVT-ensemble, we used the v-rescale thermostat (55) with a coupling constant of 0.5 ps to equilibrate the temperature to 300 K. For pressure equilibration to 1 bar, we did a 1 ns long simulation in the NPT-ensemble with the help of the Berendsen barostat (56) with a coupling constant of 5 ps. The pressure was coupled isotropically during peptide only simulations and semi-isotropically for simulations including lipid bilayers. During all equilibration steps, the positions of peptide backbone heavy atoms were position restrained using a harmonic potential with a force constant of 1000 kJ/mol/nm^2^. Lipids were kept in alignment with the z-axis by applying dihedral restraints and position restraints to the phosphorus atoms connecting lipid head and tail groups. Maintaining pressure and temperature during production simulations was achieved using the Parrinello-Rahman barostat (57) and v-rescale thermostat, respectively. Simulations of motion were integrated using the leap-frog integrator with a 2 fs time step while bond vibrations involving hydrogens were constrained using the LINCS algorithm (58). Long-range electrostatic interactions beyond a cutoff of 1.2 nm were treated with the PME method (59) and van der Waals interactions were smoothly switched to zero between a distance of 1.0 and 1.2 nm.

Different peptide conformations were sampled in solution during 10 ✕ 200 ns simulations. To embed the peptides in the membranes, we first placed the peptides above the membrane with a distance between membrane center and peptide COM of 4 nm and then pulled on the C14 atom of Myr with a velocity of 0.1 m/s until the distance between the lipid head group's phosphorous atoms and the Myr carboxy-carbon equaled zero in z-direction. The peptide-membrane systems were simulated for 1 μs under equilibrium conditions. Force-probe simulations were started from the last frame of these equilibrium simulations. The NME-cap heavy atoms were moved away from the membrane upper leaflet parallel to the z-axis at 0.1 m/s velocity and with a spring constant of 500 kJ/mol/nm^2^. For umbrella sampling, we extracted frames along the pulling trajectories with a distance of 0.2 nm and simulated them for 200 ns, reducing the pull rate to 0.0 m/s to keep a constant peptide-membrane distance during each umbrella window. The potential of mean force was computed using g_wham (60). Peptide helicity during simulations under equilibrium conditions was analyzed using the Gromacs tool gmx dssp (61) and local membrane curvature during equilibrium and force-probe simulations was determined using g_lomepro_static (62).

### Protein expression and purification

Src and Abl variant proteins were expressed using the Baculovirus system. Abl kinase variants were expressed without the C-terminal disordered region and F-actin binding domain, Uniprot_ID_P00519-2 (Abl1B, amino acids M1-K531) whereas Src was expressed as a full-length protein, Uniprot_ID_P12931-1 (Src1, amino acids M1-L536). All Src and Abl kinase variants containing a C-terminal 6 x Histidine-tag that can potentially be cleaved using TEV protease were cloned into pFastBacDualM vector. SF9 cells were transfected with pFastBacDualM-Src vector DNA using X-tremeGENE HP DNA Transfection Reagent (Roche Diagnostics) followed by cultivation in Sf-900 II SFM (1x), Serum Free Medium (Gibco by Thermo Fisher Scientific). After virus amplification an expression culture was started for each kinase variant (27 °C, 140 rpm,72 h). Cells were harvested by centrifugation (5 min, 2000 rpm, 20 °C), snap frozen in liquid nitrogen and stored at −80 °C. Thawed SF9 cell pellets were resuspended in lysis buffer (50 mM Tris pH 8.0, 500 mM NaCl, 10% Glycerol, 20 mM Imidazole, 1 mM DTT (Dithiothreitol), 1 mM PMSF (Phenylmethylsulfonylfluoride), Protease inhibitor cocktail tablet (Roche). After addition of nuclease, cell suspension was subjected to sonication (3 × 20 s) on ice. Lysates were cleared by ultra centrifugation (330,00×*g*, 4 °C, 1h) and loaded on a 5 ml Ni-column, washed with wash buffer (25 mM Hepes pH 7.4, 500 mM NaCl, 2% Glycerol, 0.5 mM DTT, 30 mM Imidazole). Src and Abl proteins were eluted using a step gradient with increasing imidazole concentrations (60/90/180/330/600 mM). Src and Abl proteins eluted at 330 mM imidazole. Fractions containing Src or Abl proteins were pooled, concentrated in 30K Amicon ultra filter concentrators and further purified by size exclusion chromatography (Superdex 200 pg HiLoad 16/600, GE healthcare) in size exclusion chromatography C buffer (25 mM Hepes, 300 mM NaCl, 5% Glycerol, 1 mM DTT, pH 7.4).

### Proteolytic digestion and peptide cleanup

Purified proteins, in 25 mM Hepes–KOH (pH 7.4), 300 mM NaCl, 5% glycerol, and 1 mM DTT, were used at the indicated concentrations and total amounts: Src WT (0.084 mg/ml, 16.8 μg), Src G2A (0.432 mg/ml, 16.8 μg), Abl WT (0.068 mg/ml, 13.6 μg), and Abl G2A (0.081 mg/ml, 16.2 μg). Bovine serum albumin (fraction V; Carl Roth) was added as a carrier protein to adjust the total protein amount to 50 μg per sample. As a positive control, a commercially available myristoylated EGFR peptide (N-myristoyl-RKRTLRRL; Cayman), dissolved in DMSO (Sigma), was added at 1950 pmol (2.55 μg). The samples were denatured in 6M urea (Sigma) and simultaneously reduced with 5 mM neutral tris(2-carboxyethyl)phosphine (Thermo Fisher Scientific) and alkylated with 40 mM 2-chloroacetamide (Thermo Fisher Scientific) for 30 min at 37 °C. 50 mM sodium phosphate pH 6.9 was added to dissolve urea to 0.8 M. Trypsin/LysC mix (Promega) was added at 25:1 protein:enzyme ratio (w/w) followed by overnight digestion at 37 °C. The digestion was stopped by acidification with trifluoroacetic acid (TFA; Sigma) to pH ≤ 2. The peptides were desalted using CDS Empore C18-HD StageTips (CDS Empore), 200 μl, 2-layers. StageTips were conditioned sequentially with methanol (Thermo Fisher Scientific), 80% acetonitrile (ACN; Merck) containing 0.1% TFA, and 0.1% TFA. Acidified peptide samples were loaded onto the StageTips, washed with 0.1% TFA, and eluted twice with 80% ACN containing 0.1% TFA. Desalted peptides were dried in a vacuum concentrator at 45 °C for 50 min. Prior to MS analysis, dried peptides were resuspended in 45 μl 2% ACN containing 0.1% TFA followed by sonication for 3 min.

### Mass spectrometry and data analysis

Peptides were analyzed using a Dionex Ultimate 3000 ultra-high-performance liquid chromatography system (Thermo Fisher Scientific), equipped with a Hypersil gold peptide C18 reversed-phase column (150 mm × 2.1 mm, 1.9 μm, 175 Å; Thermo Fisher Scientific), coupled to a Q Exactive hybrid quadrupole-Orbitrap mass spectrometer (Thermo Fisher Scientific). Mobile phases consisted of water with 0.1% formic acid (Thermo Fisher Scientific, solvent A), and acetonitrile with 0.1% FA (solvent B). Flow rate was set to 0.4 ml/min and column oven temperature was 55 °C. Peptides were separated using the following gradient of solvent B (time [min], % B): 0 min, 1%, 1.5 min, 8%, 18.5 min, 30%, 23.1 min, 60%, 27.3 min, 70%, 27.5 min, 90%, 27.9 min, 90%, 28.0 min, 1%, 28.4 min, 90%, 28.8 min, 1%, 29.3 min, 90%, 29.7 min, 0%, 30.0 min, 1%, and 34.0 min, 1%. The Q Exactive mass spectrometer was operated in positive ion mode using a data-dependent acquisition method. Full MS spectra were acquired over an m/z range of 300 to 1800 m/z at a resolution of 140,000, with AGC target of 3e6, and a maximum injection time of 200 ms. MS/MS spectra were acquired by scanning the 5 most intense precursor ions with an isolation window of 1.2 m/z, at a resolution of 17,500, with HCD normalized collision energy of 27, an AGC target 1e5 and max. Injection time of 250 ms. Precursor ions with unassigned charge states and charge states >8 were excluded. The electrospray ionization source was operated using the following settings: sheath gas 35, aux gas 10, sweep gas 0, spray voltage 3.5 kV, S-lens RF level 50, aux gas temperature 250 °C, capillary temperature 250 °C. 3 μl of resuspended peptide samples was injected.

Mass spectra were analyzed with PMI-Byos (www.proteinmetrics.com) software version 6.0.33 (Protein Metrics Inc.) using the integrated PTM identification and quantification workflow. Precursor mass tolerance was set to 5 ppm, and fragment mass tolerance to 20 ppm. Two missed cleavages were allowed for each peptide, and digestion specificity was set to fully specific. N-terminal methionine loss and cysteine carbamidomethylation were set as fixed modifications. Following variable modifications were set: myristoylation of the N-terminal glycine and arginine; methionine oxidation; carbamylation of the N-terminus and cysteine; acetylation of the N-terminus; ammonia loss of the N-terminus, cysteine and asparagine and deamidation of asparagine. A maximum of two modifications per peptide were allowed. The peptides were identified by searching the acquired MS/MS spectra against FASTA files containing the amino acid sequences of the respective proteins.

### Large unilamellar vesicle preparation

LUVs were prepared as described before (63). In brief, lipids were purchased from Avanti Polar Lipids. 1-palmitoyl-2-oleoyl-glycero-3-phosphocholine (POPC) and lissamine rhodamine B-labelled PE (16:0; Rhodamine-PE) are synthetic products, cholesterol was from ovine wool, phosphatidylinositol-4,5-bisphosphate (PI(4,5)P_2_) was derived from porcine brain. Lipid mixtures were prepared in chloroform. A gentle nitrogen stream and vacuum for 1.5 h was used to dry lipids in a round bottom flask. The dried lipid film was dissolved in buffer A (150 mM KCl, 25 mM Hepes, pH 7.4, 10% (w/v) sucrose) at 55 °C to form liposomes with a final lipid concentration of 4 mM. After transfer to 1.5 ml safe-lock tubes, 10x freeze/thaw cycles were performed in liquid nitrogen and 55 °C water bath. Finally, LUVs were subjected to 21 size extrusion steps through a 400 nm filter membrane (mini-extruder; Avanti Polar Lipids). Liposome size was determined by dynamic light scattering (DynaPro NanoStar, Wyatt Technology), with a diameter of 225 nm.

### Biochemical interaction

Liposomes with different lipid compositions (a. 99 mol% POPC + 1 mol% Rhodamine-PE, b. 10 mol% PI(4,5)P_2_ + 89 mol% POPC + 1 mol% Rhodamine-PE, or c. 10 mol% PI(4,5)P_2_ + 30 mol% Cholesterol + 59 mol% POPC + 1 mol% Rhodamine-PE) were washed with buffer B (25 mM Hepes, 150 mM KCl, pH 7.4) and sedimented (150,00*g*, 20 °C, 10 min). Following incubation with Src (1.6 μM) or Abl (1.1–2.5 μM) proteins for 2h at 25 °C, while shaking 600 rpm, membrane bound material was separated from unbound fraction by centrifugation (150,00*g*, 20 °C, 10 min). After LUVs were washed twice with buffer A, LUV pellets were resuspended in 1x reducing SDS-sample buffer. 80 to 100% of bound, 20% of unbound, and 20% of input, was loaded on a 4 to 12% Bis-Tris gel, 1.5 mm and analyzed under reducing conditions using 1x MES buffer. Coomassie stained gels were scanned in a LICOR Odyssey system. Protein signals were quantified and analyzed using Image Studio (www.licorbio.com/image-studio) Software Version 5.2.5.

### Preparation of supported lipid bilayer

SLBs composed of either pure POPC or POPC supplemented with 10% (v/v) PIP_2_ (Avanti Research, C_25_H_58_N_3_O_19_P_3_) were prepared using the lipid film hydration method. POPC (1 mg/ml) and PIP_2_ were dissolved in chloroform and the solvent was gently evaporated using nitrogen to form a thin lipid film at the bottom of a glass vial. Then the glass vial was placed under vacuum for at least 15 min to remove residual solvent. The dried films were then rehydrated in a 20 mM MOPS buffer (120 mM NaCl, 20 mM MgCl_2_) and sonicated for 30 min at room temperature. The resulting suspension was extruded through a 0.1 μm polycarbonate membrane (Cytiva) to obtain unilamellar vesicles.

### AFM imaging

Freshly cleaved mica disks were incubated overnight at 4 °C with the vesicle suspensions to allow spontaneous vesicle fusion and SLB formation. After incubation, the surfaces were thoroughly rinsed with a 20 mM MOPS buffer (120 mM NaCl) to remove unfused vesicles. Thereafter, recombinant Abl/Src kinases and their mutants (100 nM) were incubated on those surfaces for 1h at room temperature. Protein distribution on SLBs was imaged using AFM (Bruker Multimode 8) operated in tapping mode. The cantilever was oscillated at 0.5 kHz. AFM images were recorded at a resolution of 256 × 256 pixels. The peak force setpoint was maintained between 100 to 300 pN to minimize the perturbation of the sample. Bruker SNL-10 cantilever with a nominal spring constant of 0.1 N/m was used for all measurements. AFM images were analyzed using NanoScope Analysis software 2.0 (Bruker).

## Data availability

The MS proteomics data have been deposited to the ProteomeXchange Consortium *via* the PRIDE (64) partner repository with the dataset identifier PXD083765.

## Supporting information

This article contains supporting information.

## Conflict of interest

The authors declare that they have no conflicts of interest with the contents of this article.
