# Supplementary material for: Intrinsically disordered regions of Src and Abl kinases contribute to membrane binding
Source: J Biol Chem. 2026 Sep 30;302(11):113614. doi: 10.1016/j.jbc.2026.113614 (PMC13737754; doi:10.1016/j.jbc.2026.113614)
Supplement: Supplementary file 1 [file mmc1.pdf]

SI: Intrinsically disordered regions of Src and Abl kinases contribute to membrane binding

Svenja de Buhr, Julia Steringer, Irem Demir, Saber Boushehri, Ankita Ray, Christina Goss, Andrea M. Adler, Hans-Michael Müller, Rabea Goetz, Camilo Aponte-Santamaria, Britta Brügger, David Alsteens, Walter Nickel, Frauke Gräter

Supplemental Figures

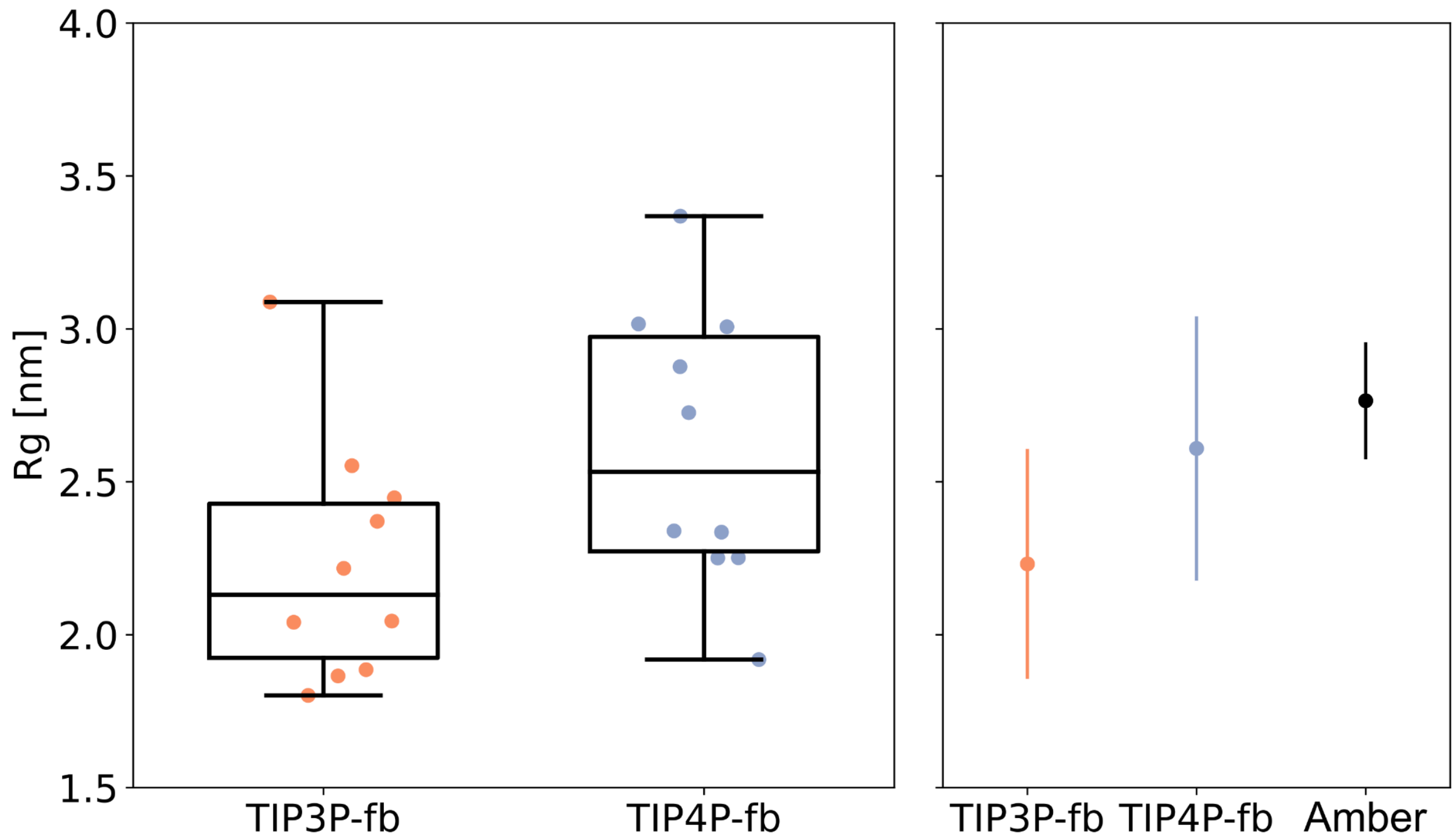

Suppl. Figure 1. Radius of gyration of the IDR of yeast transcription factor Ash1 (residues 420 - 500). RG values obtained from simulations with the Charmm36m force field in combination with TIP3P-fb or TIP4P-fb water are compared to previously published values obtained using Amber99sb-ILDNP and TIP4P-D water*[24]

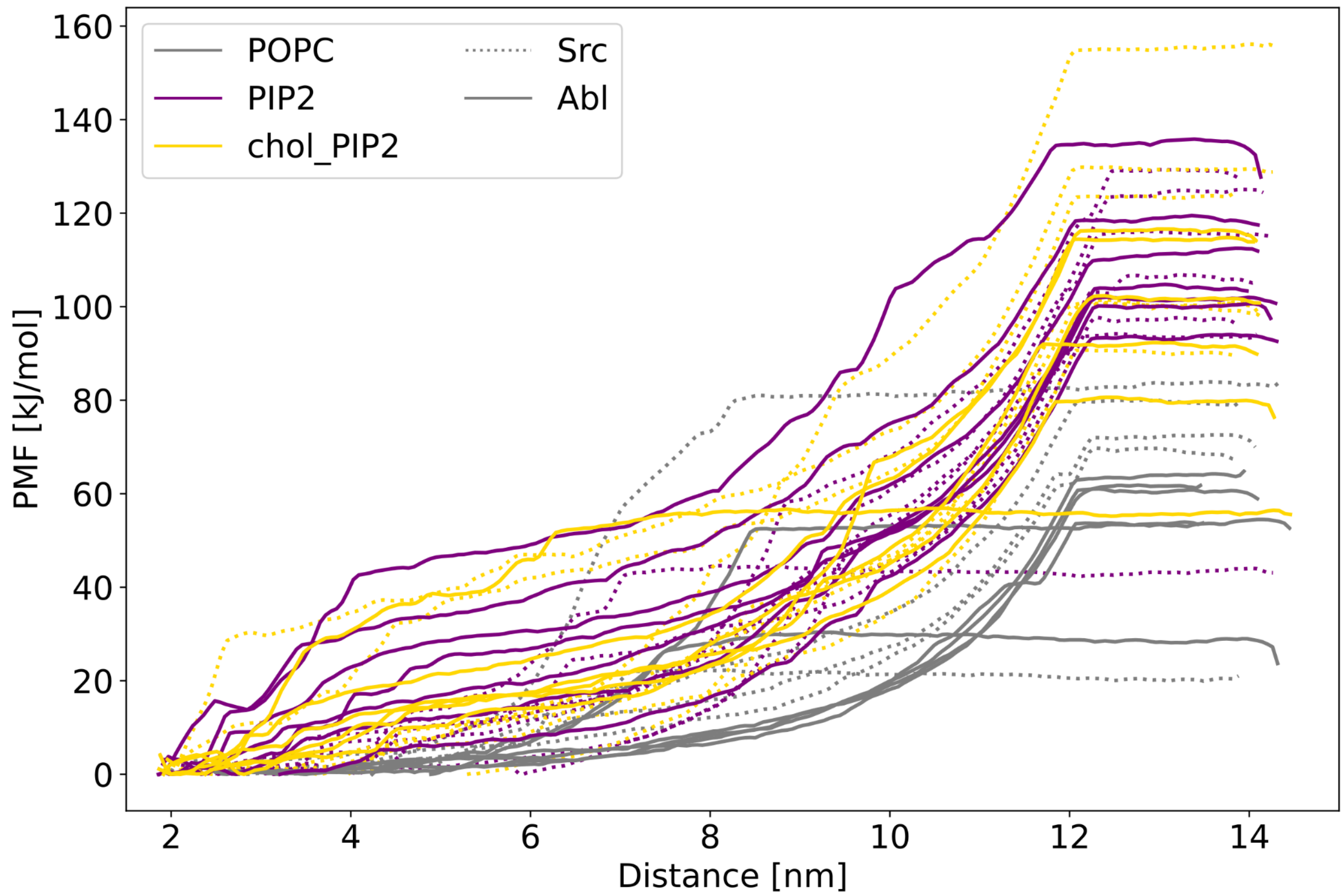

Suppl. Figure 2. Potential of mean force (PMF) along the distance between lipid bilayers and myristoylated N-terminal peptides of Src and Abl.

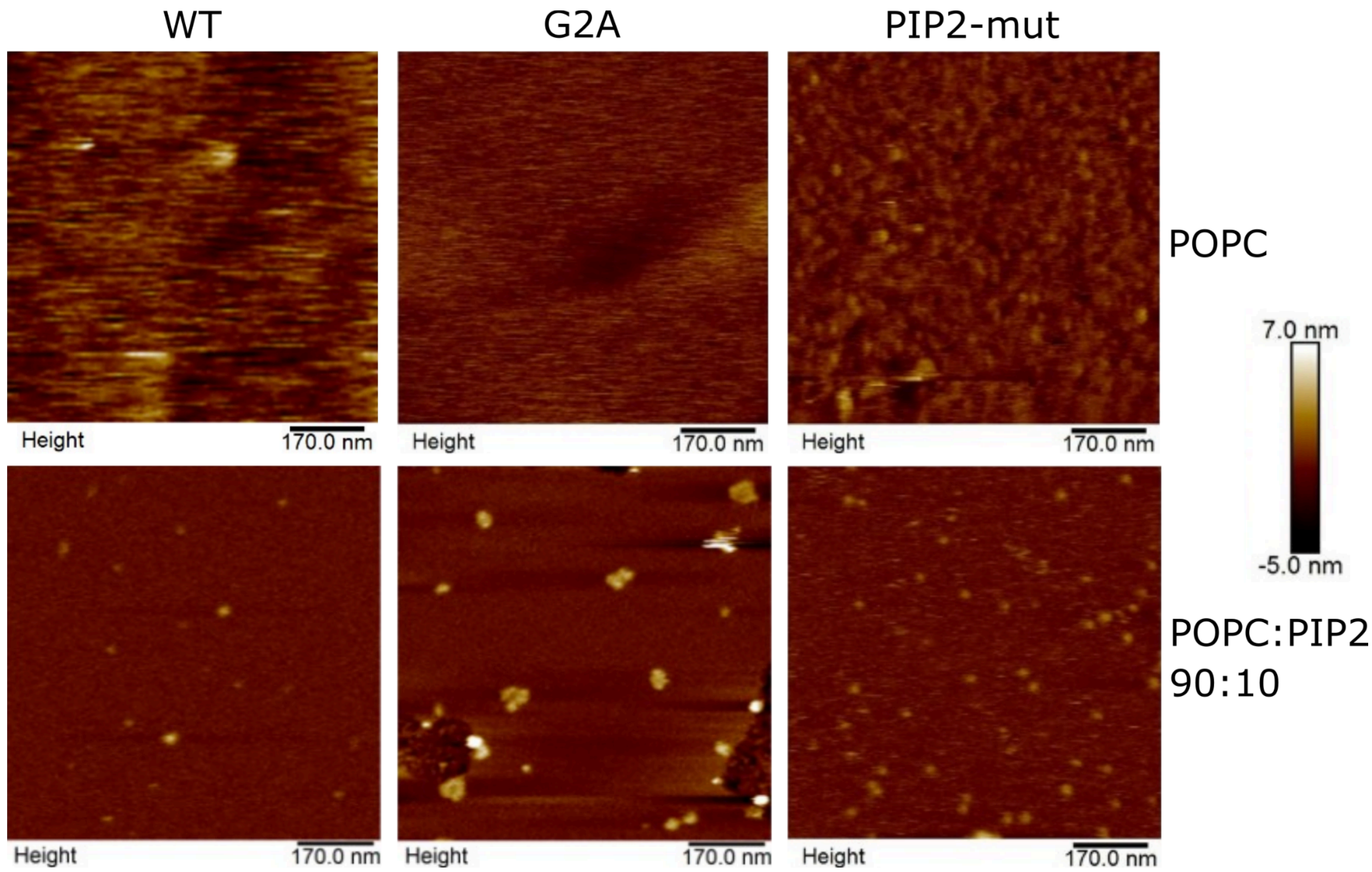

Suppl. Figure 3. AFM images of Src on a pure POPC membrane (top) or a POPC membrane with 10% PIP_2. The protein was in the wt state (left, corresponds to figure 5B), had a mutation that prohibited myristoylation (G2A, middle), or positively charged residues where mutated to Gln (PIP2, right).

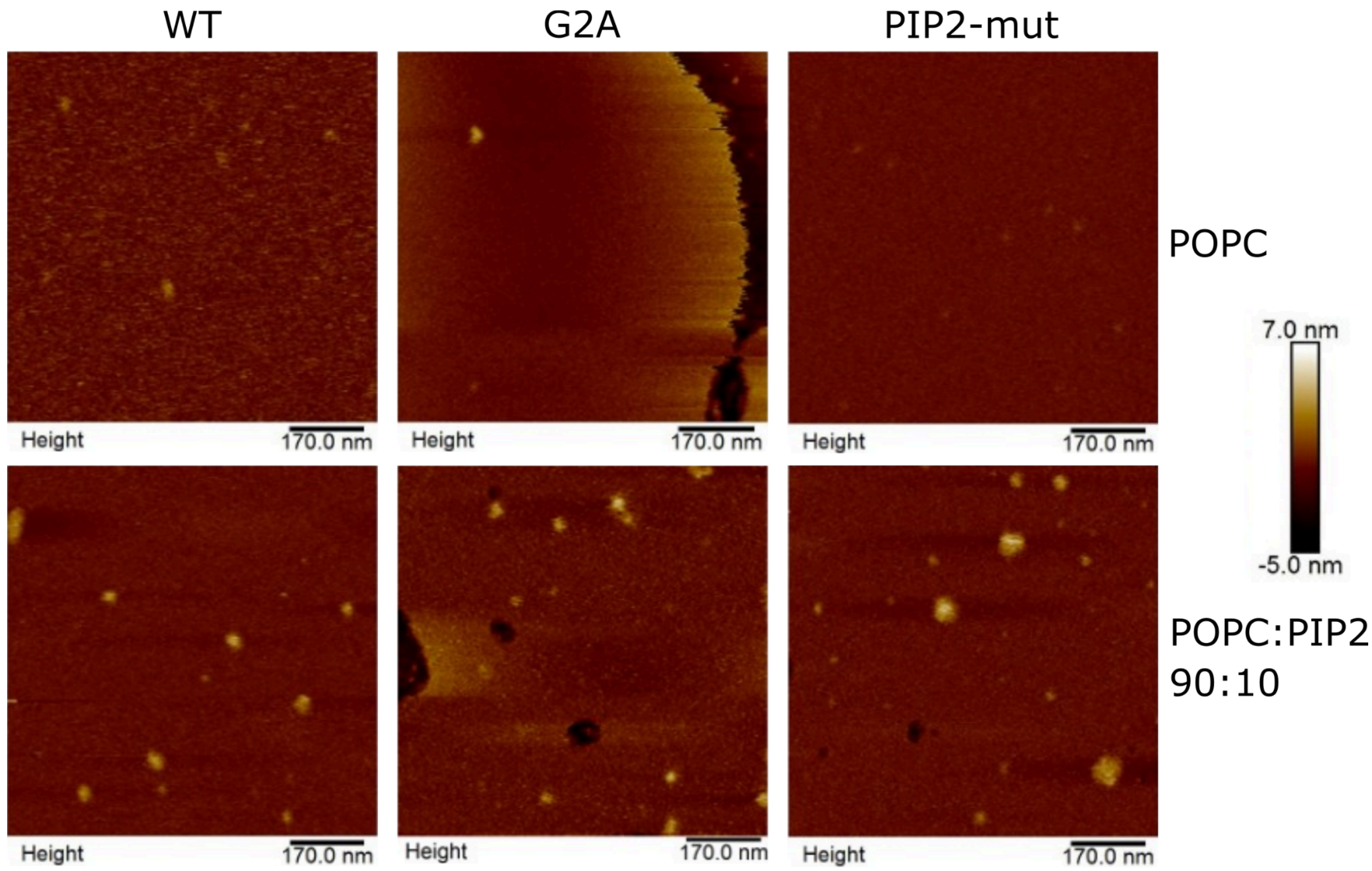

Suppl. Figure 4. AFM images of Abl on a pure POPC membrane (top) or a POPC membrane with 10% PIP_2. The protein was in the wt state (left, corresponds to figure 5B), had a mutation that prohibited myristoylation (G2A, middle), or positively charged residues where mutated to Gln (PIP2, right).

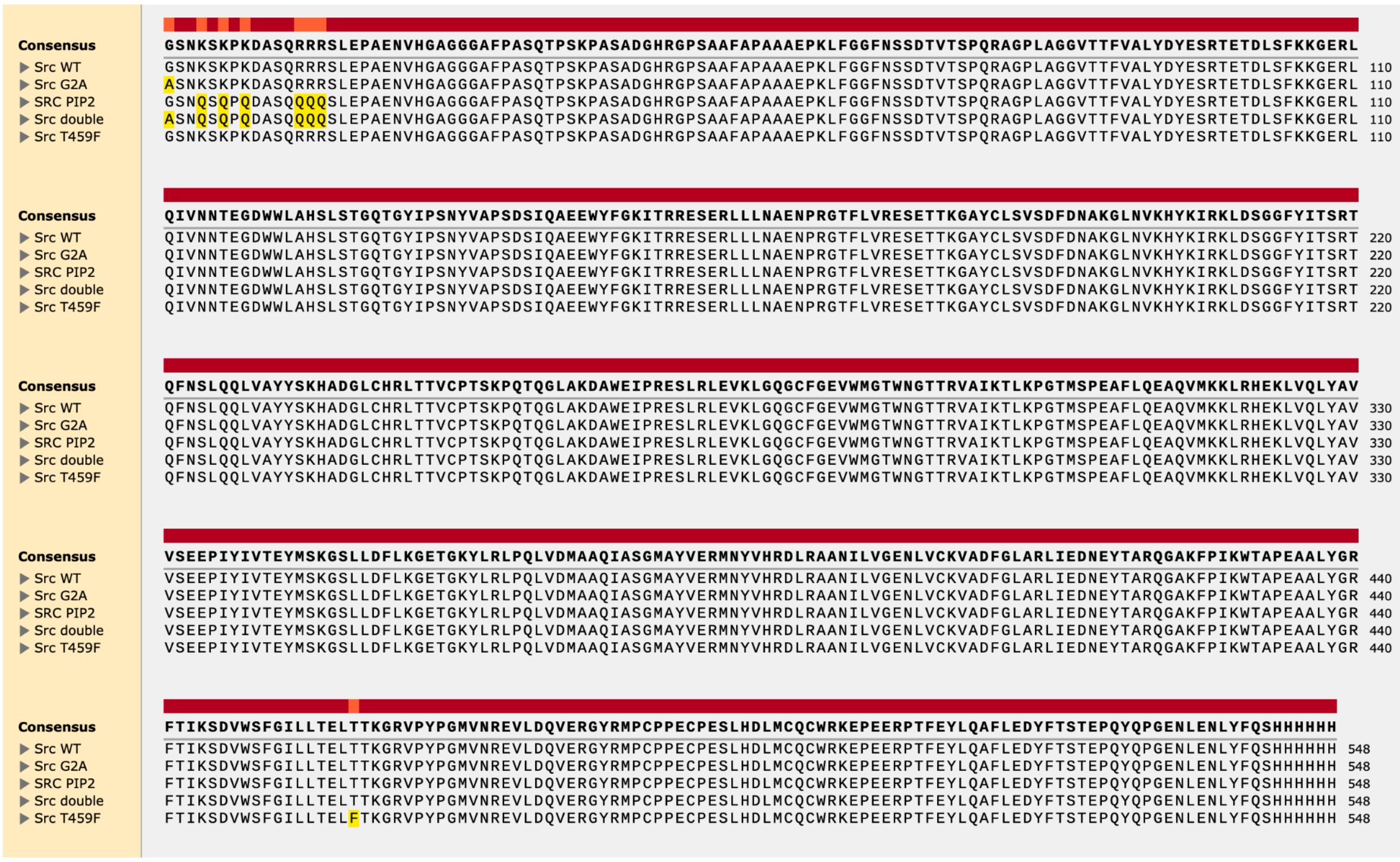

Suppl. Figure 5. Protein sequence alignment of expressed Src protein variants without start methionine highlighting amino acid exchanges (SnapGene software version 8.2.1).

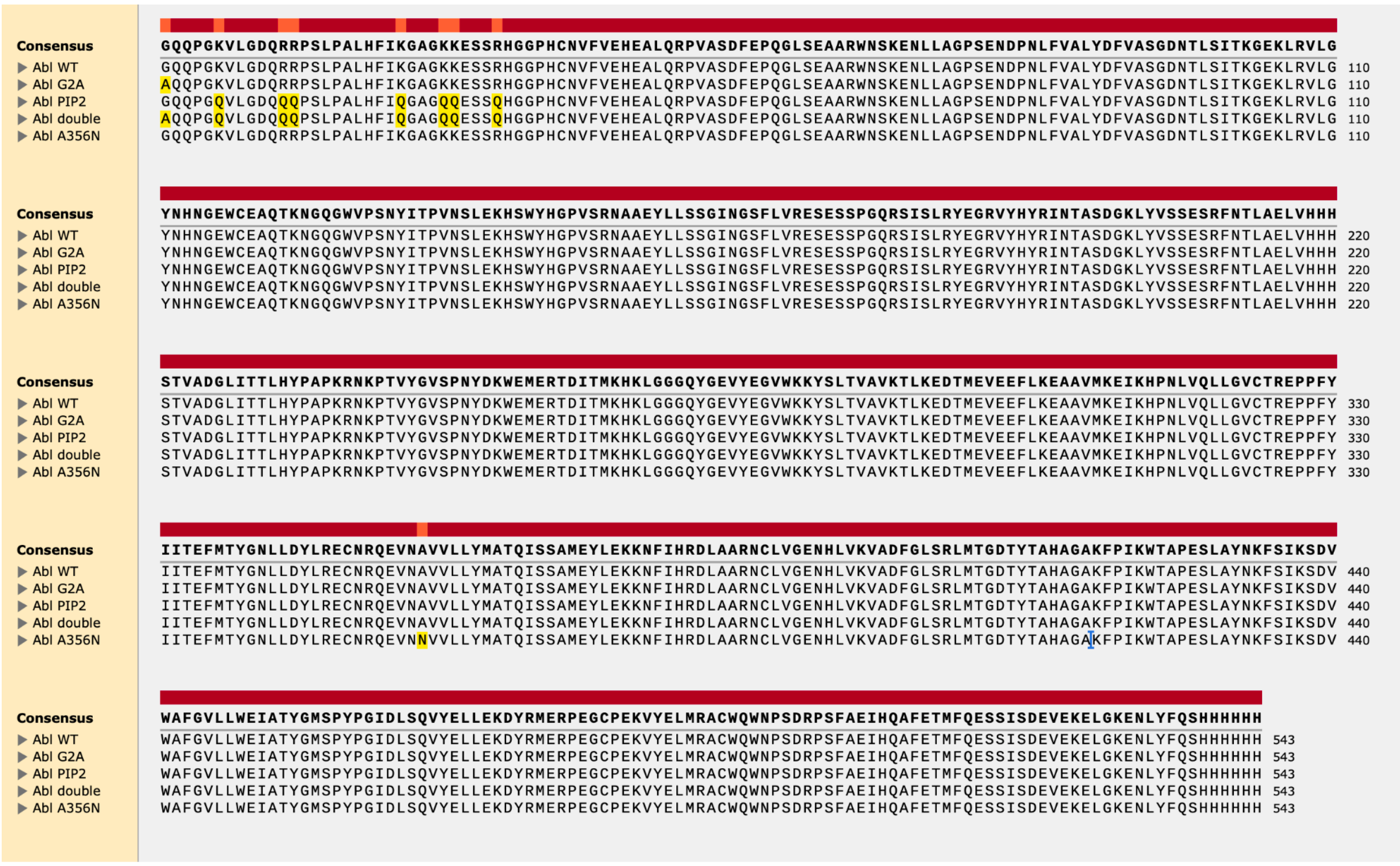

Suppl. Figure 6. Protein sequence alignment of expressed Abl protein variants without start methionine highlighting amino acid exchanges (SnapGene software version 8.2.1).

Src wildtype

Sequence coverage

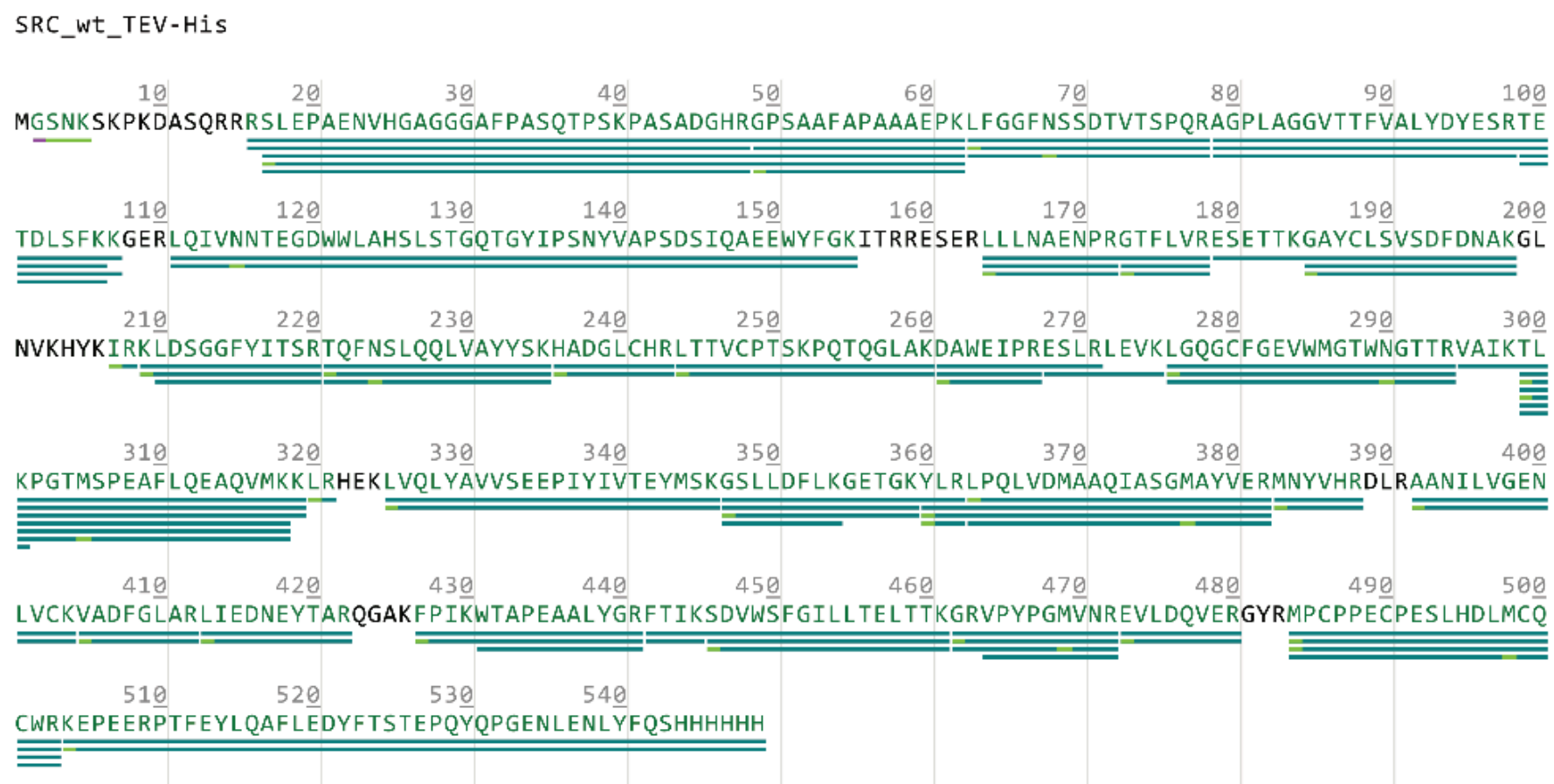

Extracted ion chromatogram (XIC)

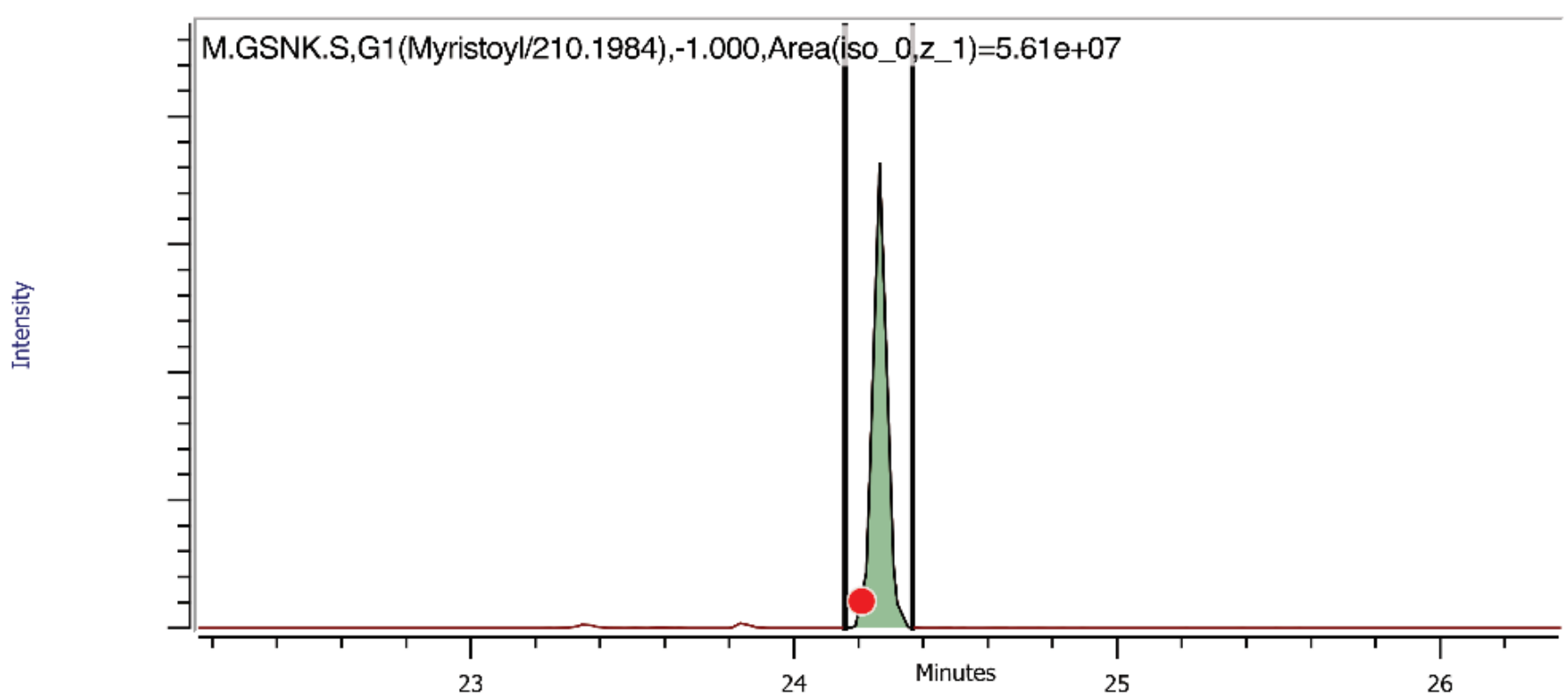

Precursor isotope pattern

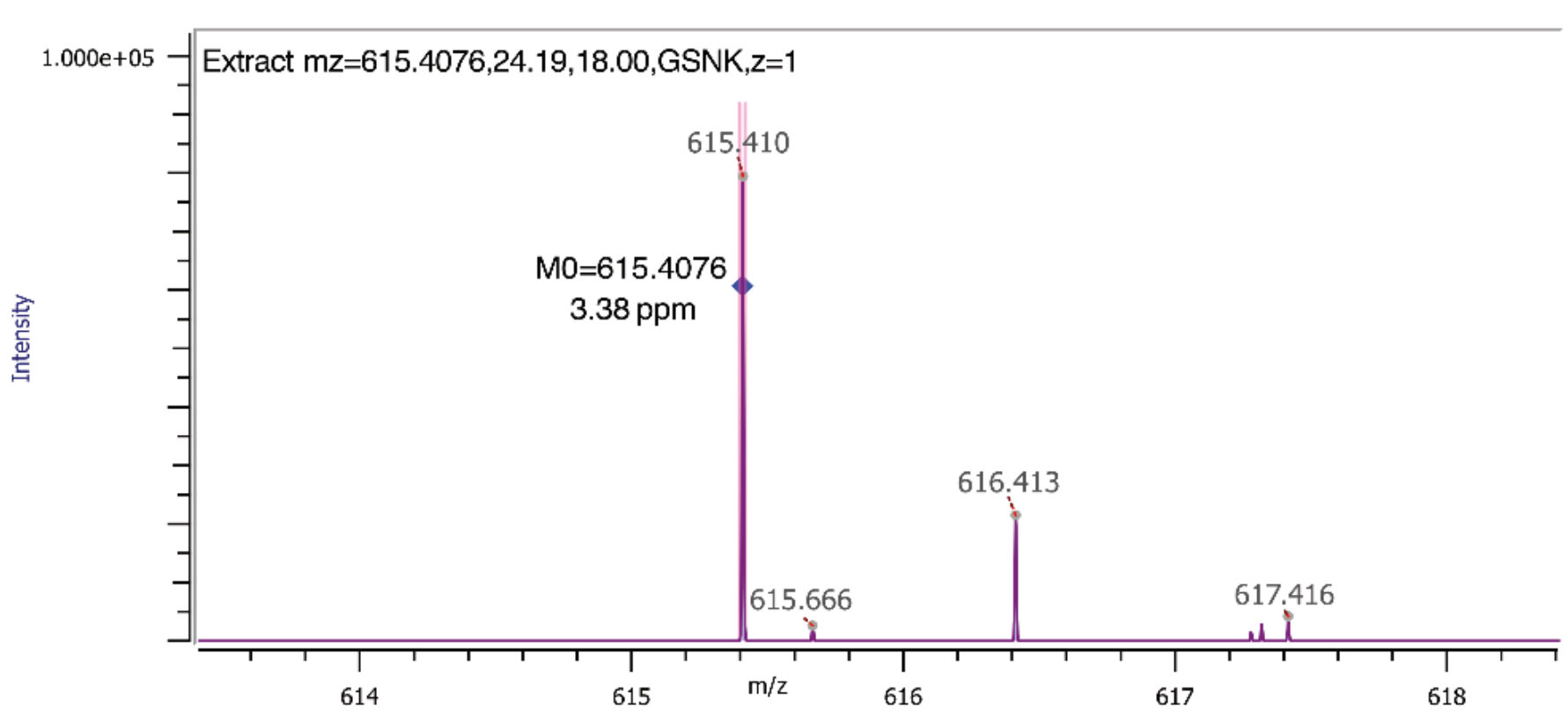

MS/MS spectrum

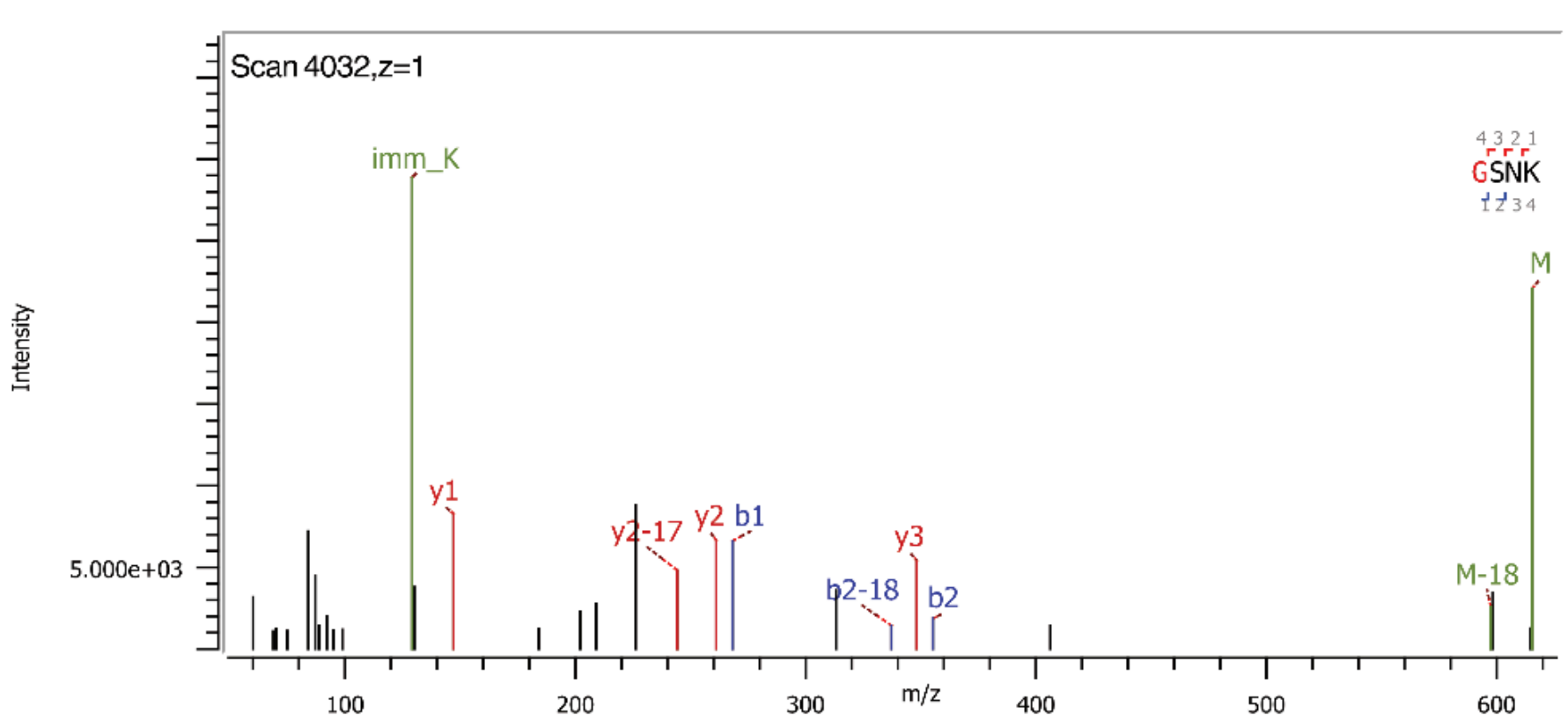

Suppl. Figure 7. LC–MS/MS identification of N-terminal myristoylation in Src wildtype. Sequence coverage, extracted ion chromatogram (XIC) of the identified N-terminal peptide, precursor isotope pattern, and corresponding MS/MS spectrum are shown. The N-terminal peptide GSNK of Src wildtype was identified in its N-myristoylated form. In the sequence coverage plots, the N-terminal peptide of interest is highlighted in neon green, with the myristoylated amino acid indicated in purple. Other identified peptides are shown in dark green and additional modified residues are indicated in light green. n=1

Abl wildtype

Sequence coverage

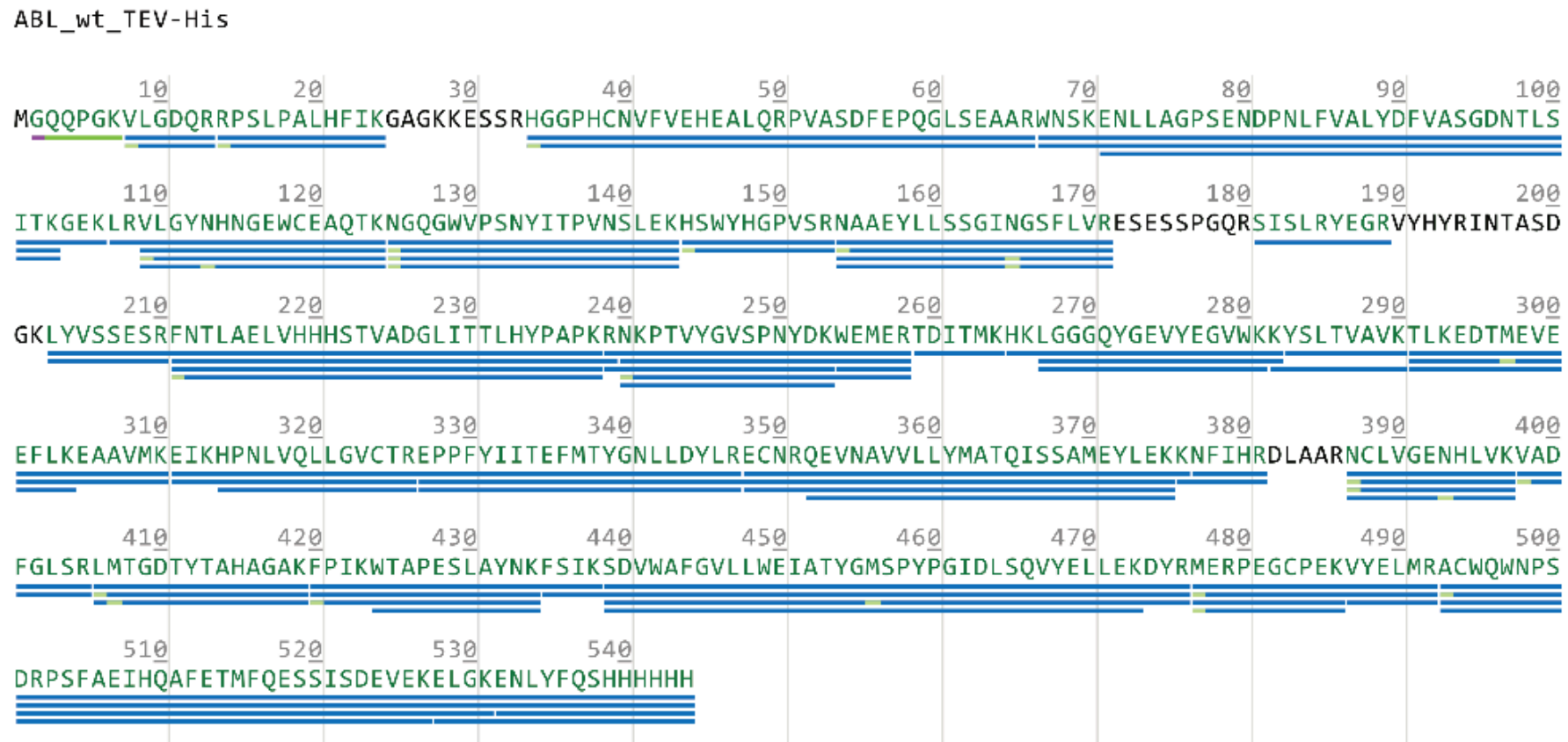

Extracted ion chromatogram (XIC)

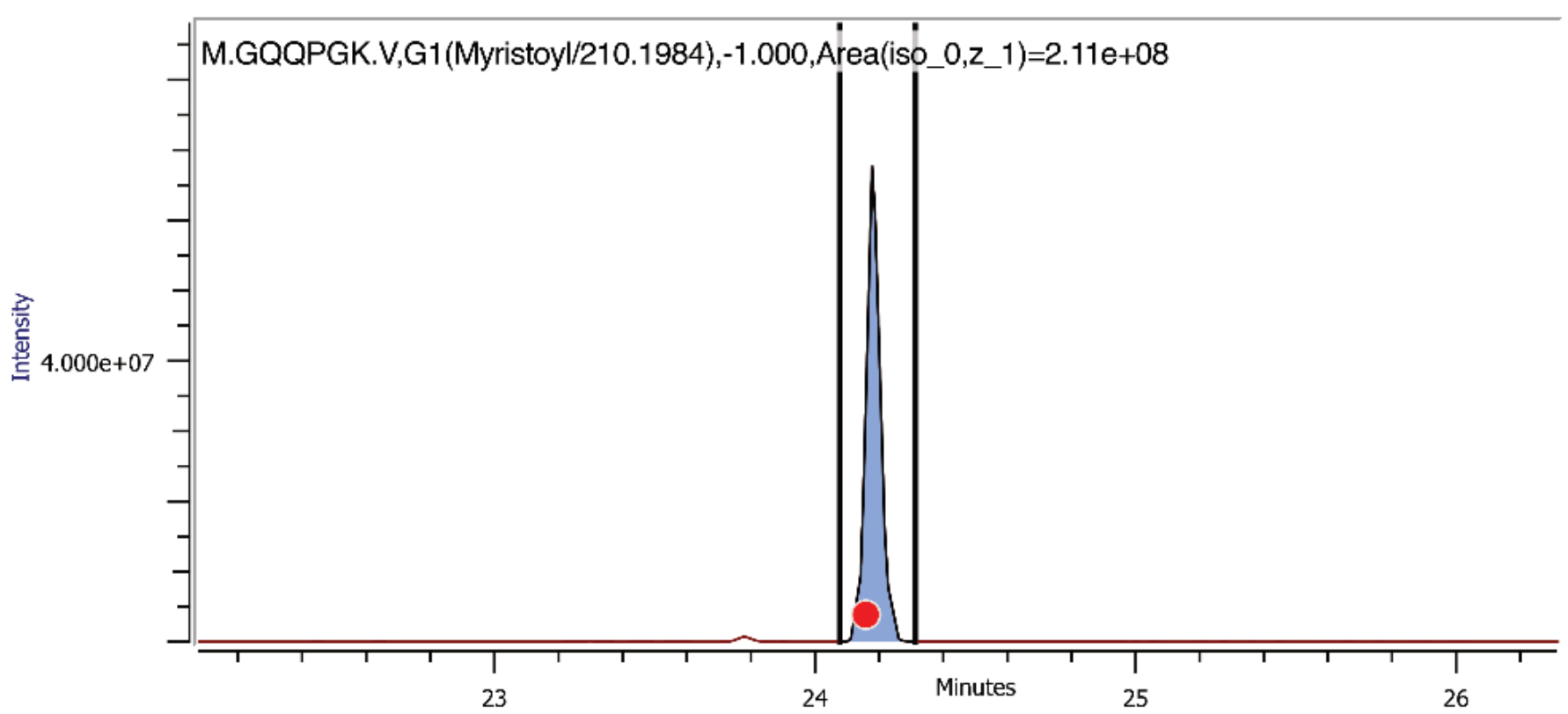

Precursor isotope pattern

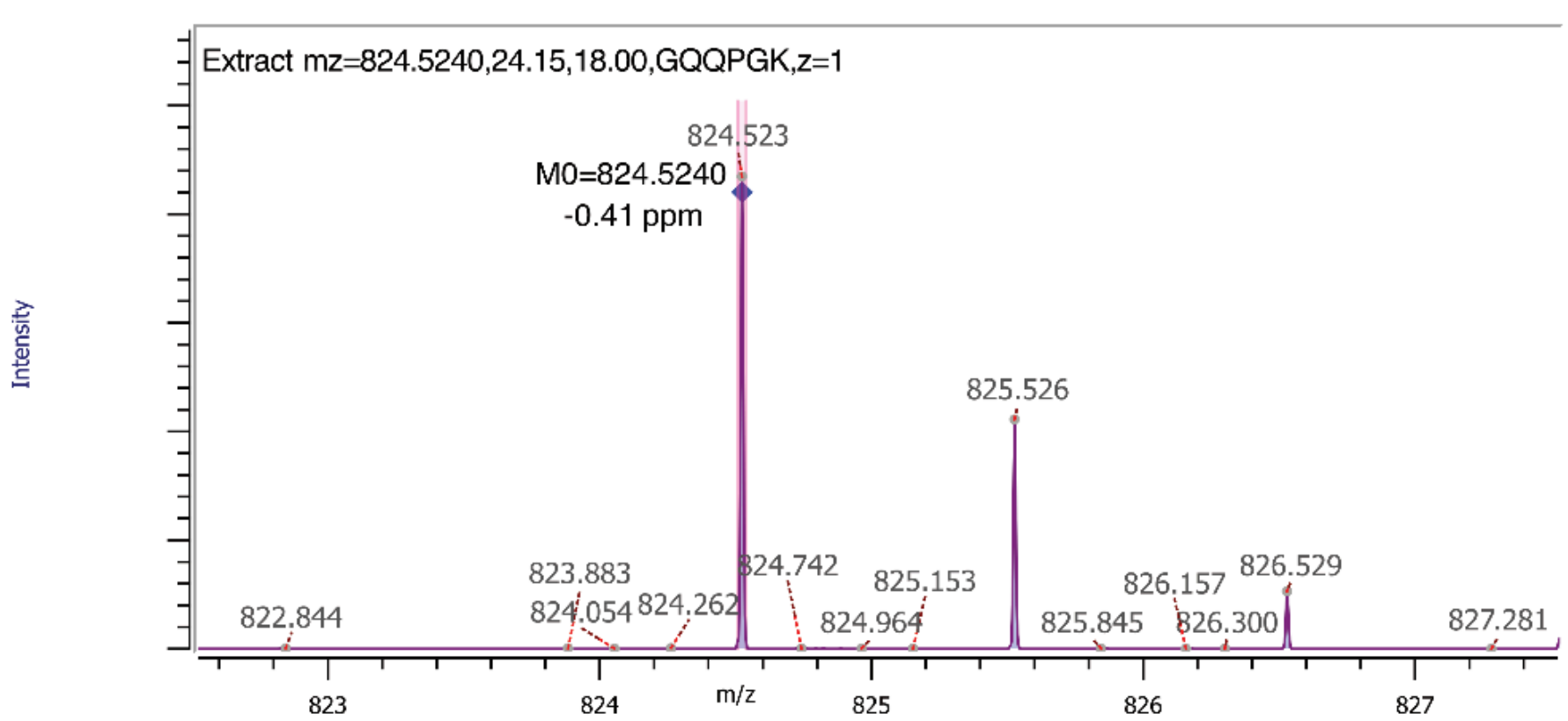

MS/MS spectrum

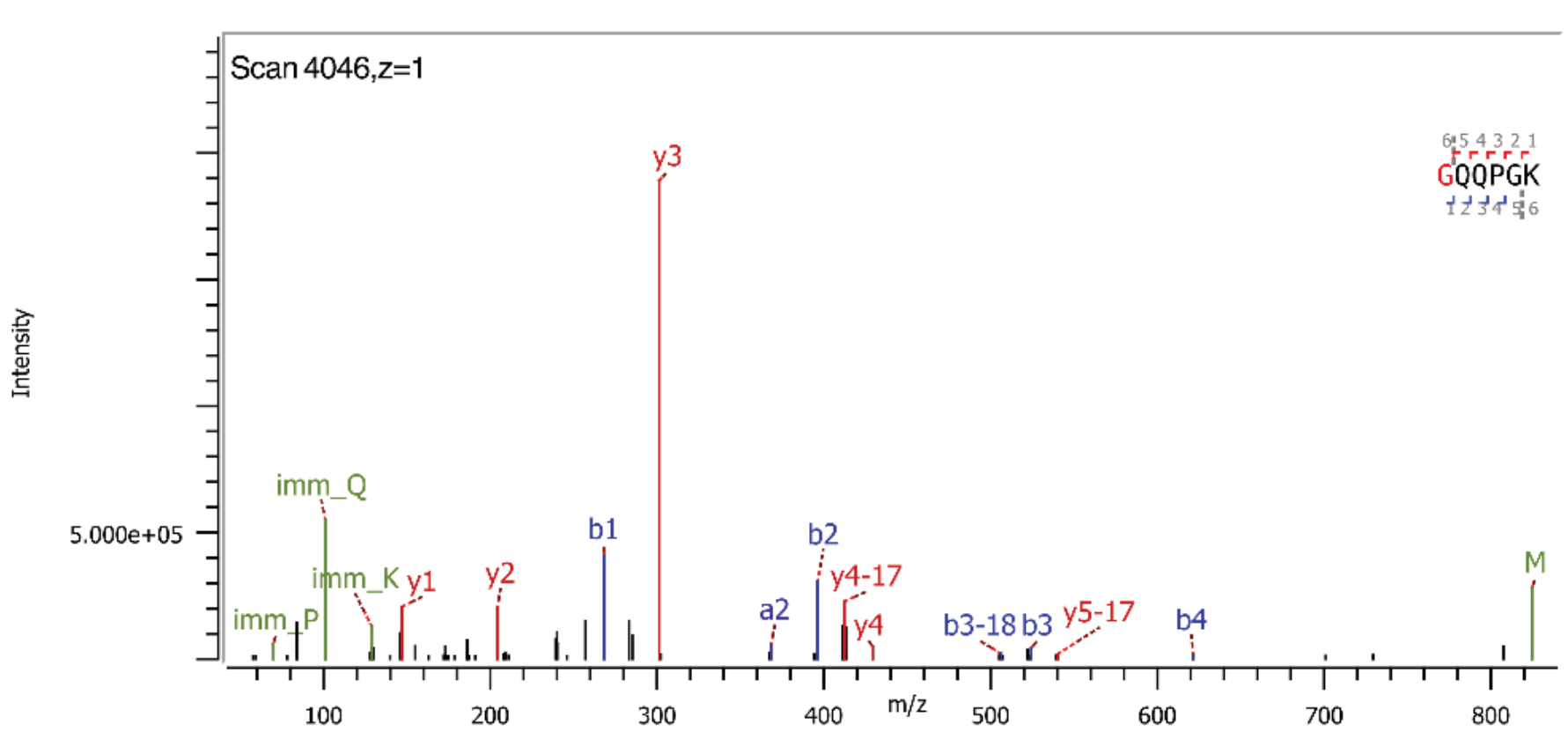

Suppl. Figure 8. LC–MS/MS identification of N-terminal myristoylation in Abl wildtype. Sequence coverage, extracted ion chromatogram (XIC) of the identified N-terminal peptide, precursor isotope pattern, and corresponding MS/MS spectrum are shown. The N-terminal peptide GQQPGK of Abl wildtype was identified in its N-myristoylated form. In the sequence coverage plots, the N-terminal peptide of interest is highlighted in neon green, with the myristoylated amino acid indicated in purple. Other identified peptides are shown in blue and additional modified residues are indicated in light green. n=1

Abl G2A

Sequence coverage

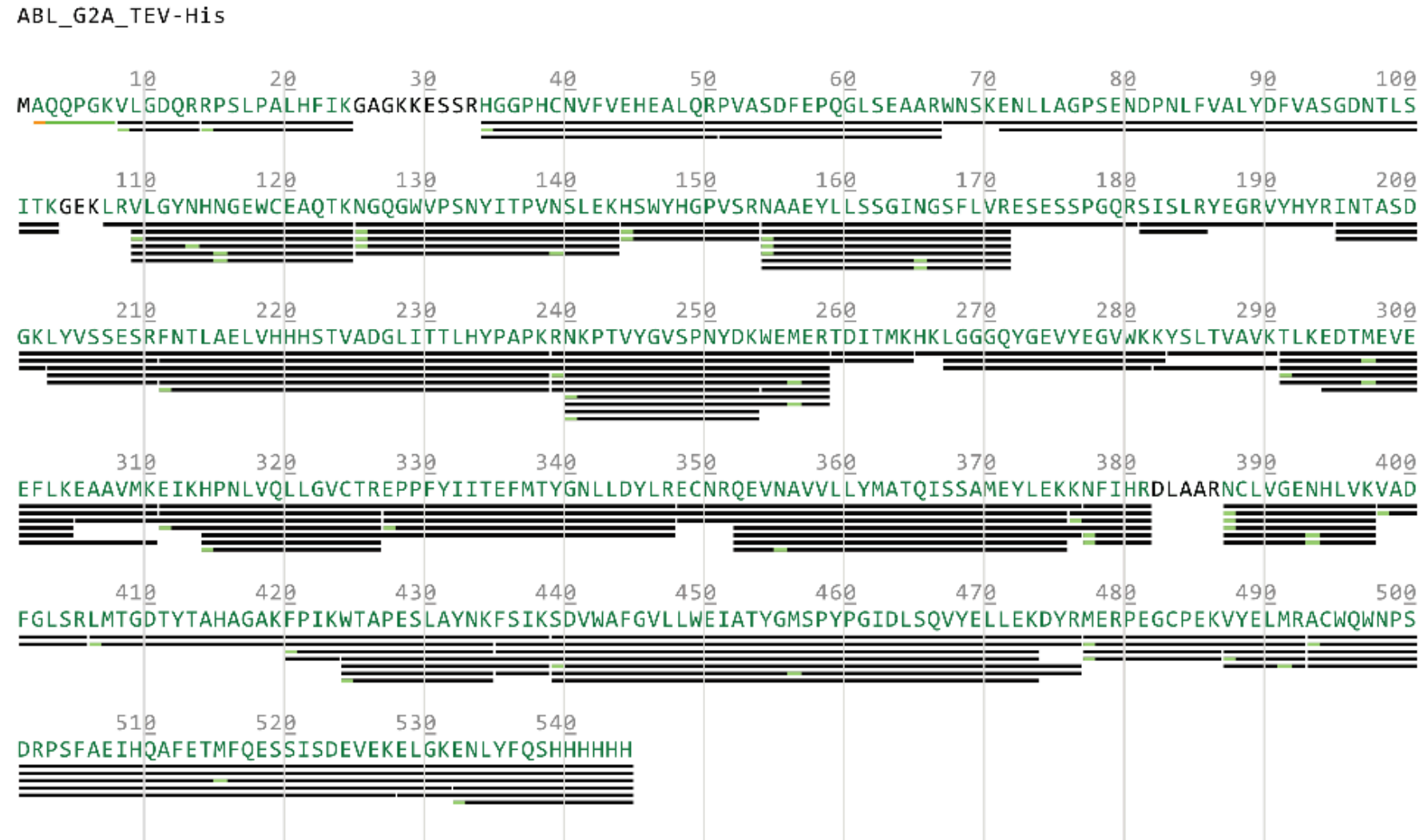

Extracted ion chromatogram (XIC)

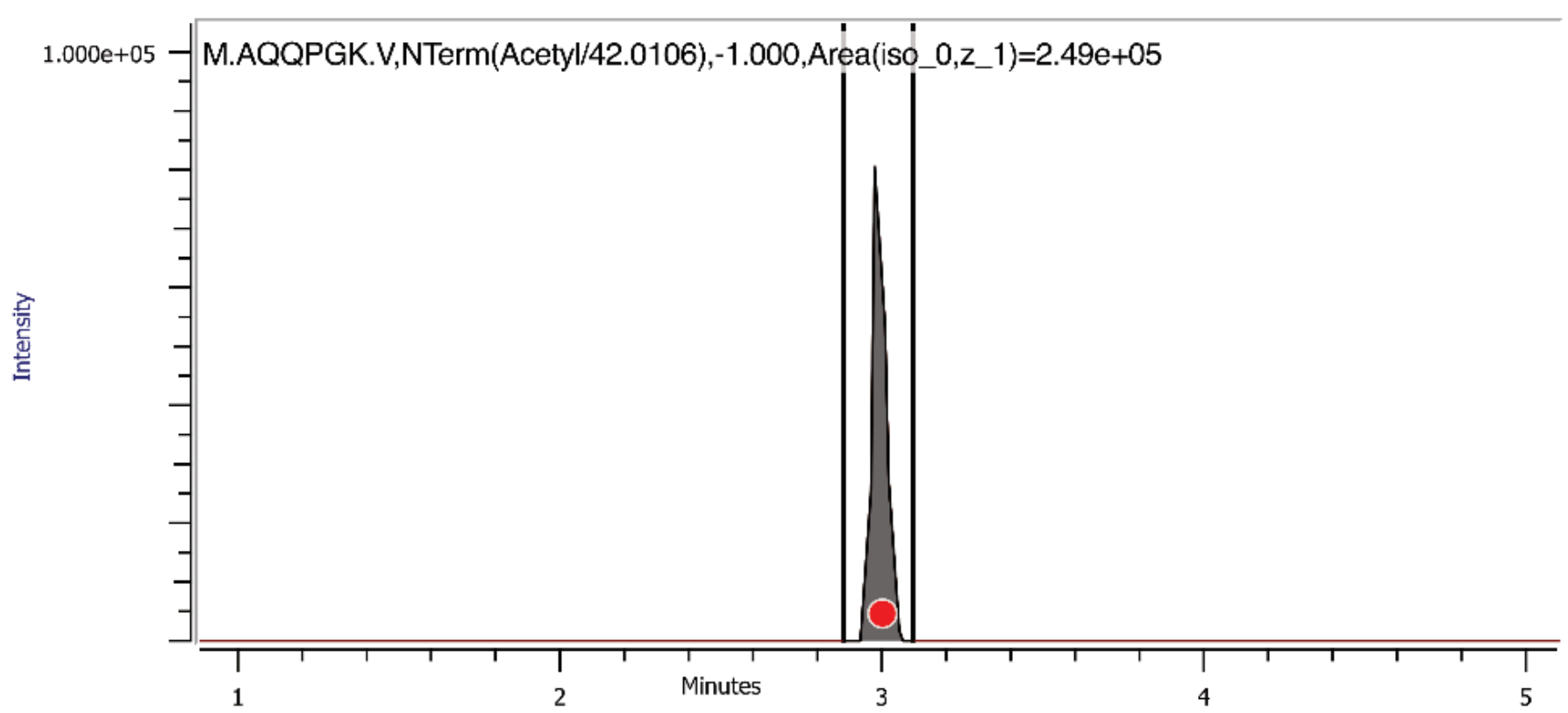

Precursor isotope pattern

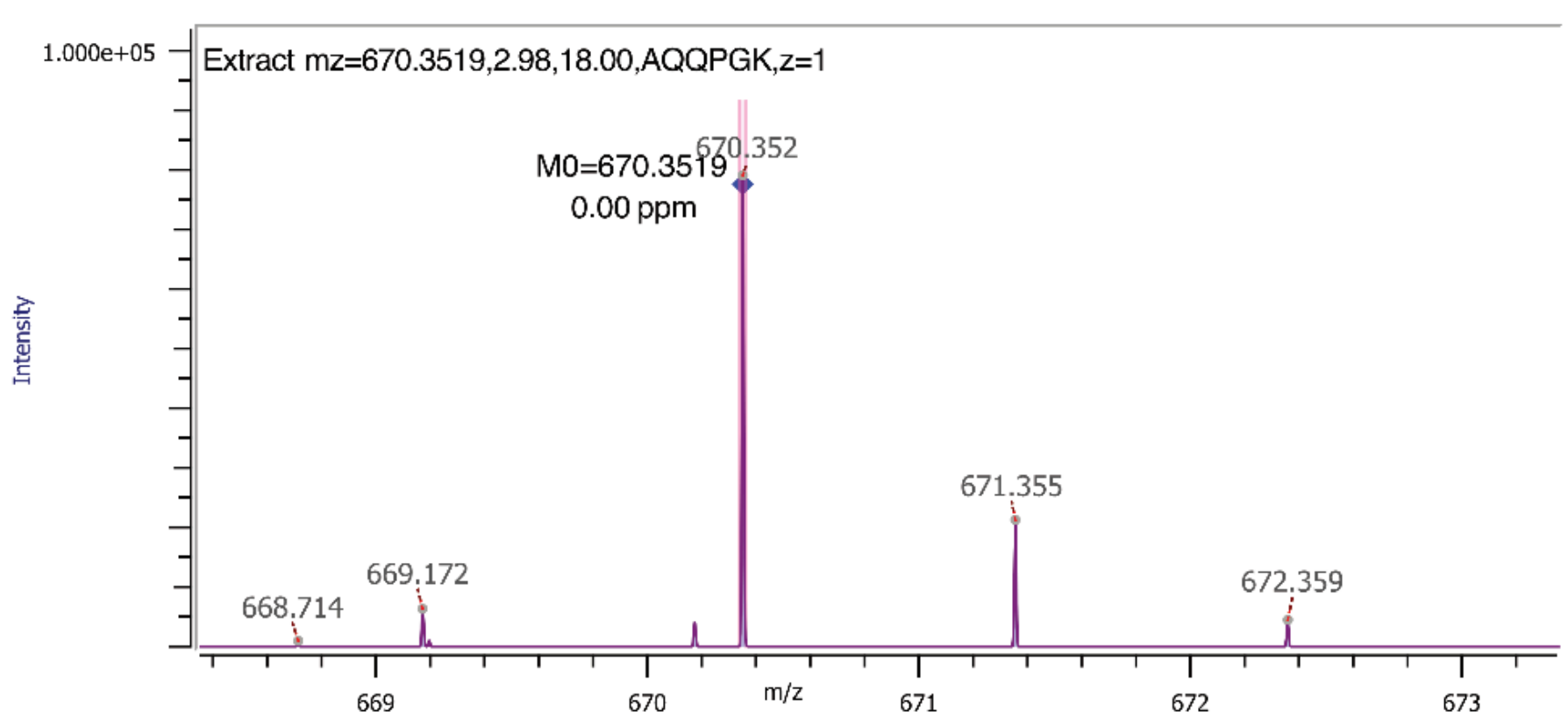

MS/MS spectrum

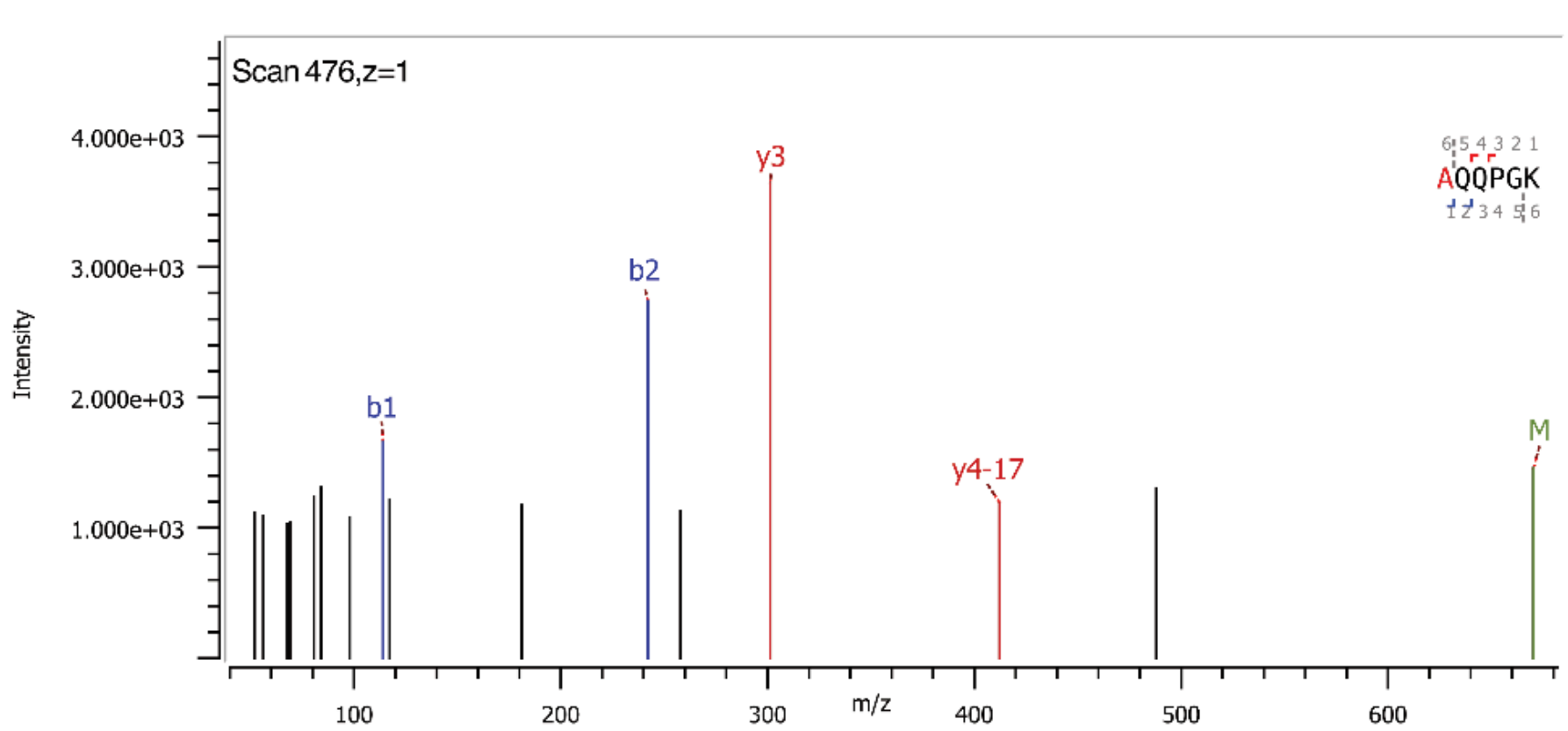

Suppl. Figure 9. LC–MS/MS identification of N-terminal peptide in Abl G2A. Sequence coverage, extracted ion chromatogram (XIC) of the identified N-terminal peptide, precursor isotope pattern, and corresponding MS/MS spectrum are shown. The N-terminal peptide AQQPGK of the Abl G2A mutant was identified in its N-terminally acetylated form. In the sequence coverage plots, the N-terminal peptide of interest is highlighted in neon green, with the acetylated amino acid indicated in orange. Other identified peptides are shown in black and additional modified residues are indicated in light green. n=1

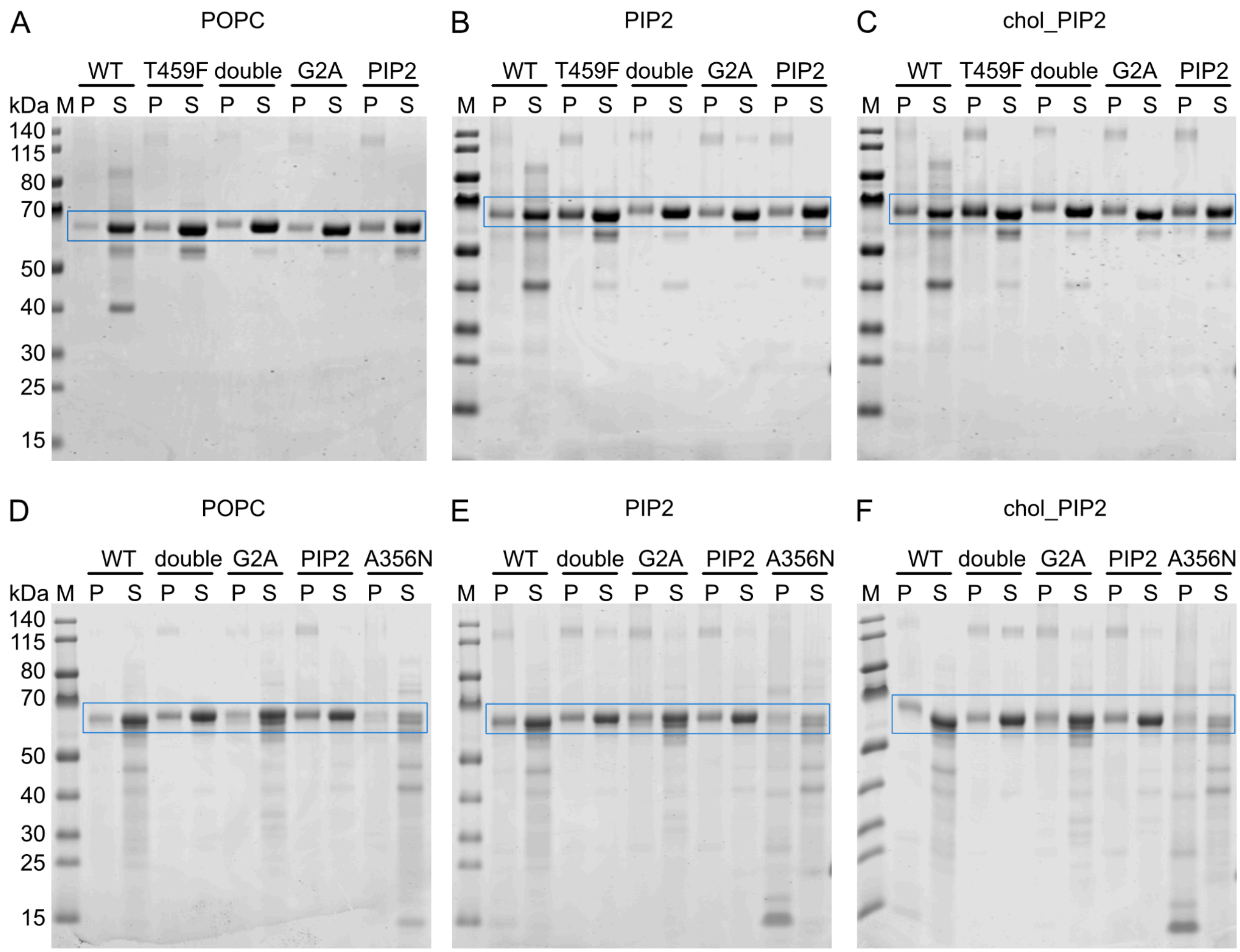

Suppl. Figure 10. Liposome Binding of Src (A-C) and Abl (D-F). Representative images of SDS-PAGE gels run under reducing conditions. 99 mol% POPC + 1 mol% Rhodamine-PE (POPC,A and D), 10 mol% PIP_2 + 89 mol% POPC+ 1 mol% Rhodamine-PE (PIP2, B and E), or 10 mol% PIP_2 + 30 mol% Cholesterol + 59 mol% POPC + 1 mol% Rhodamine-PE (chol_PIP2, C and F). Pellet (P, 100% loaded), supernatant (S, 20% loaded). Coomassie stained gels were scanned in a LICOR Odyssey system. Blue rectangles indicate expressed proteins (62 kDa).

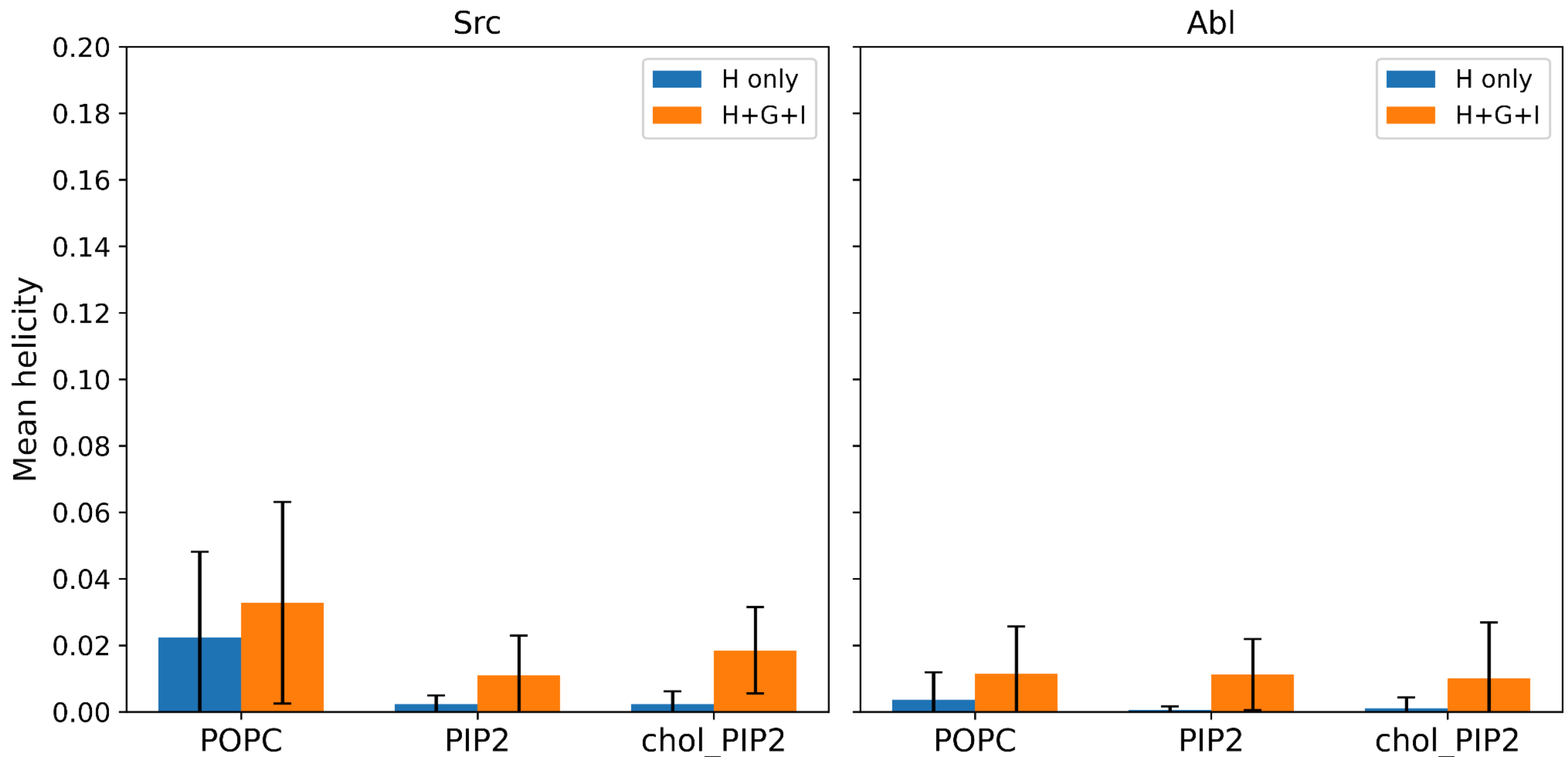

Suppl. Figure 11. Helicity of the Src and Abl N-terminal disordered region on the membrane surface. Secondary structure assignment was performed for every frame using the GROMACS DSSP algorithm. Results are derived from equilibrium MD simulations (1 µs). In these configurations, the myr-group is inserted into the hydrophobic core of the membrane, while the remaining residues extend into the aqueous phase and interact with the lipid headgroups. The figure compares the mean fraction of residues in a helical conformation for Src (left) and Abl (right) across three membrane environments: POPC, PIP2, and chol_PIP2. Two metrics are reported: H only (blue) represents pure alpha-helix content. H+G+I (red) represents total helical propensity, including alpha-helix, 3_10-helix, and pi-helix. The bars represent the mean fraction of helicity calculated across 10 independent replicas, with error bars representing the standard deviation.

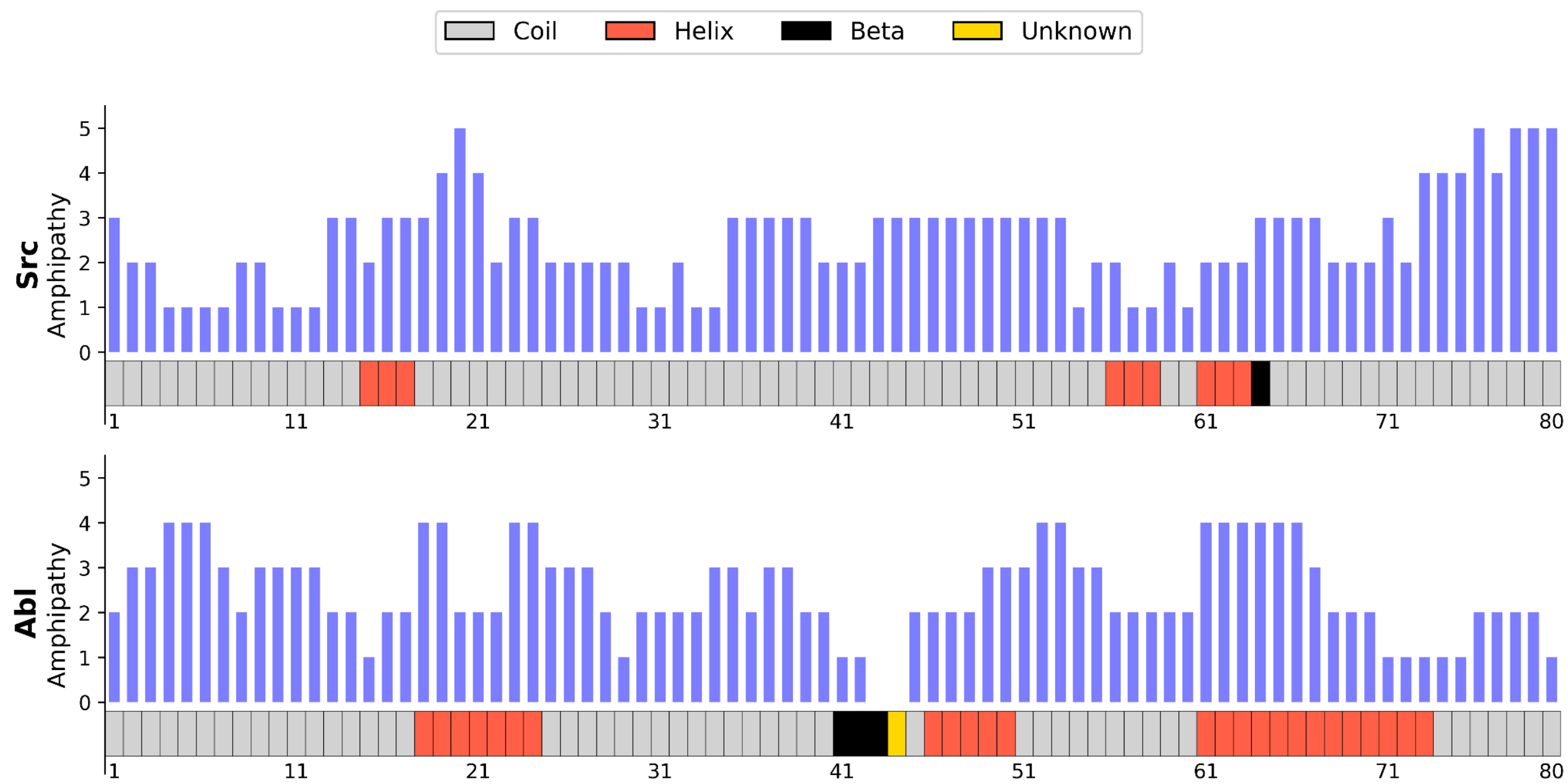

Suppl. Figure 12. Secondary structure and amphipathicity profiles for the N-terminal regions (residues 1–80) of Src and Abl. The colored horizontal strip indicates the secondary-structure prediction from AmphipaSeeK, where gray denotes coil, red denotes helix, blue denotes beta structure, and yellow denotes uncertain assignment. The blue bars show the amphipathy score for each residue position (from 0=low to 5=high amphipathy, based on the sequence average).

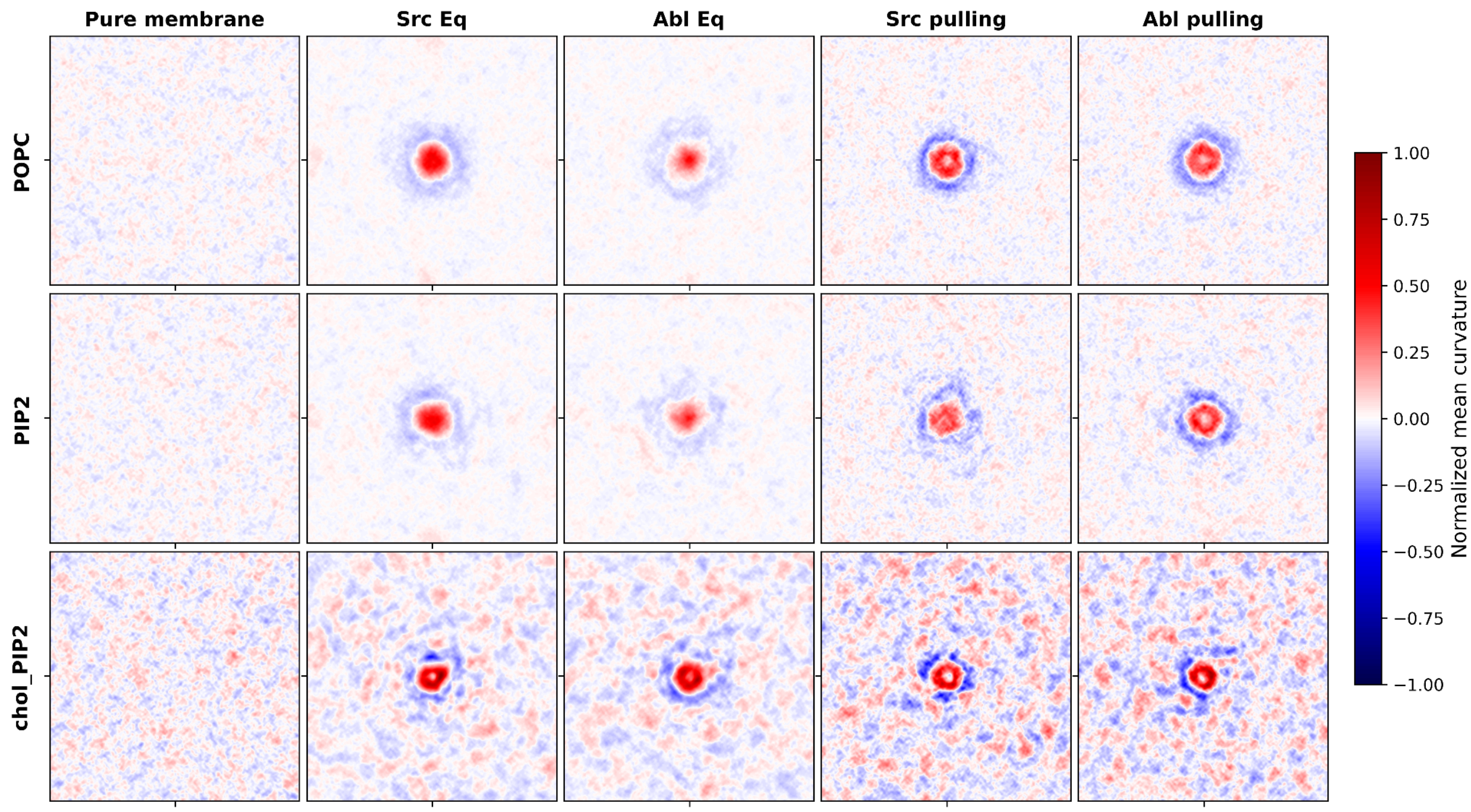

Suppl. Figure 13. Upper-leaflet membrane curvature maps for five different systems: pure membrane, Src equilibrium simulations, Abl equilibrium simulations, Src pulling simulations, and Abl pulling simulations, shown for three membrane compositions (POPC, PIP2, and chol_PIP2). Curvature values were averaged over the available replicas for each system and normalized using a single global maximum absolute value across all panels, resulting in a common scale from -1 to 1. Red indicates positive or inward bending, whereas blue indicates negative or outward bending. Pure membranes remain mostly flat. However, when the protein is present, the membrane bends inward at the center (where the myristoyl group inserts) and outward in the surrounding area.
